# 4000 years of human dietary evolution in central Germany, from the first farmers to the first elites

**DOI:** 10.1371/journal.pone.0194862

**Published:** 2018-03-27

**Authors:** Angelina Münster, Corina Knipper, Vicky M. Oelze, Nicole Nicklisch, Marcus Stecher, Björn Schlenker, Robert Ganslmeier, Matthias Fragata, Susanne Friederich, Veit Dresely, Vera Hubensack, Guido Brandt, Hans-Jürgen Döhle, Werner Vach, Ralf Schwarz, Carola Metzner-Nebelsick, Harald Meller, Kurt W. Alt

**Affiliations:** 1 Institute of Anthropology, Johannes Gutenberg University, Mainz, Germany; 2 Curt-Engelhorn-Centre for Archaeometry gGmbH, Mannheim, Germany; 3 Anthropology Department, University of California, Santa Cruz, California, United States of America; 4 Center of Natural and Cultural History of Man, Danube Private University (DPU), Krems-Stein, Austria; 5 State Office for Heritage Management and Archaeology, Saxony-Anhalt/State Museum of Prehistory, Halle/Saale, Germany; 6 Xylem Analytics Germany Sales GmbH & Co. KG, Mainz, Germany; 7 State Office for Heritage Management, Saxony, Dresden, Germany; 8 Max Planck Institute for the Science of Human History, Jena, Germany; 9 Clinical Epidemiology Group, Center for Medical Biometry and Medical Informatics, Medical Center—University of Freiburg, Freiburg, Germany; 10 Institute of Prehistoric Archaeology and Provincial Roman Archaeology, Ludwig Maximilian University of Munich, Munich, Germany; 11 Department of Biomedical Engineering and Integrative Prehistory and Archaeological Science, Basel University, Basel, Switzerland; University of Otago, NEW ZEALAND

## Abstract

Investigation of human diet during the Neolithic has often been limited to a few archaeological cultures or single sites. In order to provide insight into the development of human food consumption and husbandry strategies, our study explores bone collagen carbon and nitrogen isotope data from 466 human and 105 faunal individuals from 26 sites in central Germany. It is the most extensive data set to date from an enclosed geographic microregion, covering 4,000 years of agricultural history from the Early Neolithic to the Early Bronze Age. The animal data show that a variety of pastures and dietary resources were explored, but that these changed remarkably little over time. In the human *δ*^15^N however we found a significant increase with time across the different archaeological cultures. This trend could be observed in all time periods and archaeological cultures (Bell Beaker phenomenon excluded), even on continuously populated sites. Since there was no such trend in faunal isotope values, we were able largely to exclude manuring as the cause of this effect. Based on the rich interdisciplinary data from this region and archaeological period we can argue that meat consumption increased with the increasing duration of farming subsistence.

In *δ*^13^C, we could not observe any clear increasing or decreasing trends during the archaeological time periods, either for humans or for animals, which would have suggested significant changes in the environment and landscape use. We discovered sex-related dietary differences, with males of all archaeological periods having higher *δ*^15^N values than females, and an age-related increasing consumption of animal protein. An initial decrease of *δ*^15^N-values at the age of 1–2 years reveals partial weaning, while complete weaning took place at the age of 3–4 years.

## Introduction

Dietary evolution is fundamental to human history [1]. Throughout most of that history, people lived as hunter-gatherers and used a range of plants and animals in their natural environment. Adaptation to different habitats and ecosystems is a feature of cultural evolution, and there is no single, “natural” human diet. Approximately 12,000 years ago, at the end of the last glaciation, the process of Neolithisation started in the Near East. The beginning of agriculture and animal husbandry irreversibly resulted in the most radical change in human economic and subsistence strategies and the largest social modification in the history of humankind [2]. Food resources became more and more abundant and constant. The consumption of cereals and other plants increased dramatically [3]. What impact did farming have on human lifestyle? The domestication of plants and animals led to changes in numerous aspects of life, including available foodstuffs, physical activities, reproductive experience, psychosocial relations, microbial interactions, toxin/allergen exposure, and sedentism. In several waves, Neolithic pioneers spread along different routes from the Fertile Crescent and reached central Europe by about 6,000 cal. BC [4,5]. Ancient DNA analyses, including mtDNA and Y-chromosome analyses, and genome-wide scans, revealed that the introduction of the “Neolithic package”, including previously unknown animals and plants, agricultural economy, sedentism, ceramics and new technologies, was closely connected to a population-genetic event [6–11]. Moreover, there is also evidence for large-scale migrations at the end of the Neolithic and in the Early Bronze Age in the form of fundamental changes in the archaeological record [7,12]. These ground-breaking insights into population dynamics in central Europe raise fundamental questions regarding their impact on human subsistence strategies during the Neolithic, starting with the establishment of the food-producing economic system and ending in the Early Bronze Age.

Here we present a diachronic study that focuses on socio-economic changes, and socio-cultural aspects of those changes, over almost 4,000 years of agricultural history in the Middle Elbe-Saale region (MES) of central Germany (5,500 to 1,550 cal. BC). Our aim is to provide insight into human food consumption and husbandry strategies and to determine if, when and why people started to intensify livestock breeding and dairy farming during the Neolithic. In the 1980s, Andrew Sherratt postulated the model of a “secondary products revolution” that emphasises the discovery and systematic use of secondary animal resources, such as milk, wool, and the use of draught animals [13]. The model itself and the influence of secondary animal resources on socioeconomic changes in Neolithic cultures are still controversially discussed [14]. The results of our diachronic study can contribute powerful arguments to this discourse. We use carbon and nitrogen stable isotope analyses of human and faunal bone collagen [15–17] to reconstruct human dietary patterns. In one of the largest studies of the Neolithic and Early Bronze Age undertaken to date, the data reflect alterations in dietary composition, especially with regard to the role of animal-derived proteins in the human diet, but also the intensity of arable land cultivation. Owing to its fertile soils, the Middle Elbe-Saale (MES) region was densely settled throughout prehistory. It therefore offers excellent conditions for studying the establishment and development of the food-producing economy over time. The rich archaeological record not only documents fundamental alterations in human material culture, but also includes continuously settled sites, which allow human dietary changes to be investigated in areas, which were constantly inhabited. The MES survey addresses the following key questions regarding dietary development:

1. Did changes occur in the composition of the human diet from the Linearbandkeramik Culture (LBK), at the transition between the Mesolithic and the beginnings of the farming lifestyle, to the establishment of the Únětice Culture in the Early Bronze Age, both at individual sites and across the entire study area?
2. What diachronic trends can be observed in relation to the supply of fodder for domestic animals and husbandry strategies in general?
3. Do our data reveal indications for or against a "secondary products revolution"?
4. Does the role of animal protein from meat, fresh milk, or dairy products in the human diet change over time?
5. Do possible dietary changes correlate with the genetic population dynamics in central Germany?
6. Are there any differences in the diet between the sexes and the various age groups in the period studied?
7. Do infant weaning patterns change in the context of changing food supply?

The present study was part of an interdisciplinary research project entitled “Cultural change = Population change?”. Based on archaeological, physical anthropological (sex, age, stature, health status, stress factors), ancient DNA (settlement and population history, matri-/patrilines, kinship analysis) and stable isotope analyses, it investigated the settlement and population history of the MES in the context of information on lifestyle and life conditions [6,8,18–21]. This is the first study to outline the development of diet as part of the cultural revolution on an epochal scale from the Neolithic to the Early Bronze Age. Thanks to excellent preconditions, the MES is a key area for researching the diet and cultural development that took place after the introduction of the farming lifestyle in Europe.

## The Neolithic in central Germany

**Fig 1:**
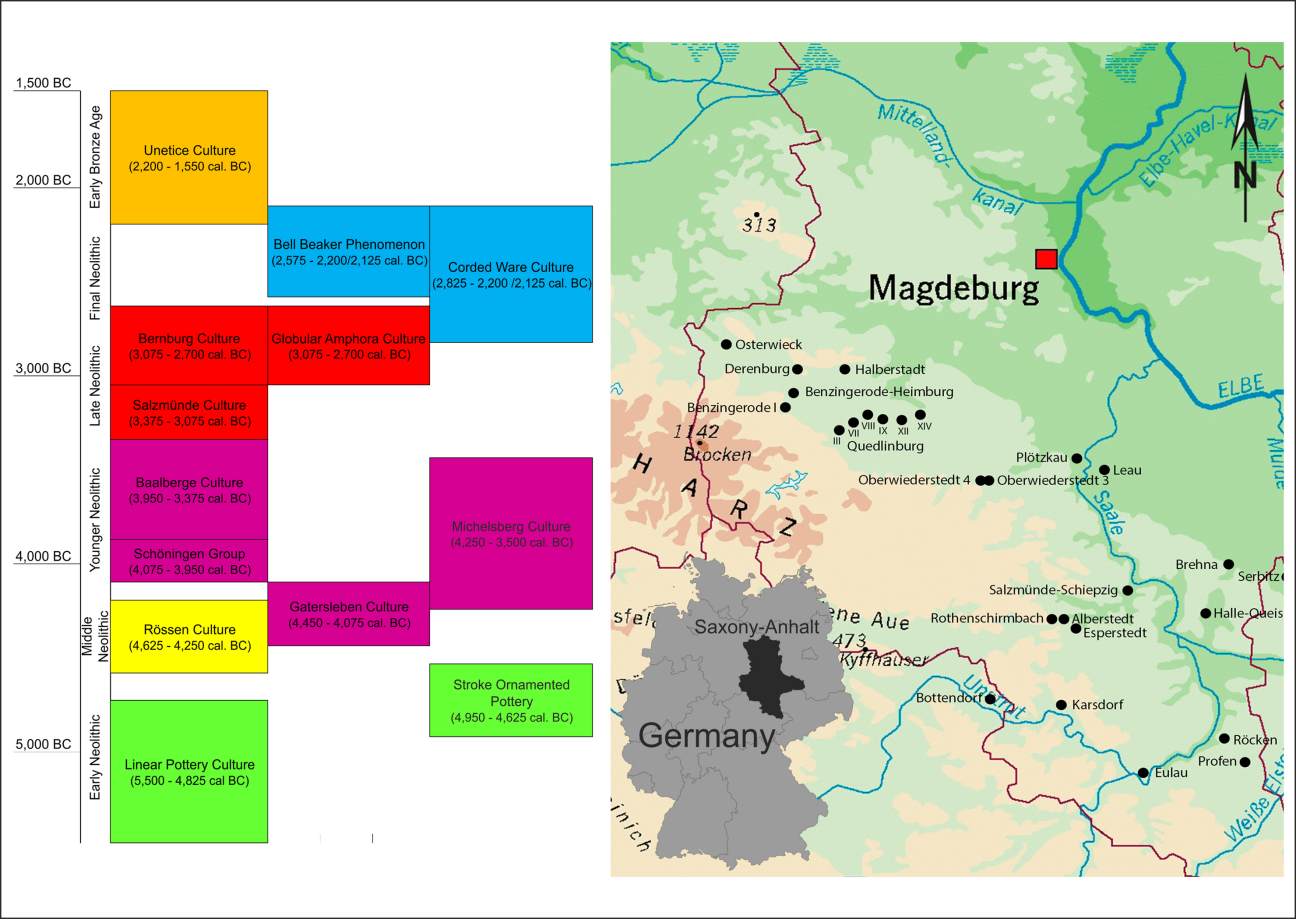
Overview of investigated sites and archaeological chronology of Neolithic and Early Bronze Age central Germany. The Stroke Ornamented Culture and Michelsberg Culture are not represented in our sample due to low rate of anthropological findings. Chronology after Schwarz in [29].

The Middle Elbe-Saale region is located at the intersection of the Elbe and Saale rivers in the south of Saxony-Anhalt, Germany. Due to its fertile soils and a rather lightly forested landscape [22,23] it provided ideal conditions for farming and animal husbandry. During the Neolithic and the Early Bronze Age, the area was settled by the bearers of numerous archaeological cultures, from the earliest farmers and cattle breeders to the metal-processing societies which saw the emergence of the first social elites. In order to achieve a representative characterisation of the dietary profiles over a timeframe of just under 4,000 years, the most promising approach appeared to be a diachronic survey divided up into six periods. The onset of the Early Neolithic (EN) in the MES is dated to c. 5,500 cal. BC and is represented by the widespread archaeological phenomenon of the Linearbandkeramik Culture (LBK). The chronological span of the Middle Neolithic (MN) covers the Rössen Culture (RSC) and is dated to c. 4,625–4,250 cal. BC. It is followed by the Younger Neolithic (YN), during which a cultural regionalisation can be observed. The YN comprises the Gatersleben Culture (GLC), the Schöninger Group (SCG) and the Baalberge Culture (BAC). The Late Neolithic (LN) begins around 3,375 cal. BC with the Salzmünde Culture (SMC), the Bernburg Culture (BEC) and the Globular Amphora Culture (KUC). This is followed from 2,825 cal. BC onwards by the Final Neolithic (FN), which includes the transregional Corded Ware Culture (CWC) and Bell Beaker phenomenon (BBC), which overlapped with each other chronologically. Finally, the Early Bronze Age (EBA), represented by the Únětice Culture (UC), begins around 2,200 cal. BC [24–28] (Fig 1). More information on the archaeological cultures is provided in S5 Table. This rich archaeological record represents supra-regional as well as regional cultural phenomena and chronological trends. Thanks to a firm association of cultural groups with periods by archaeological means and statistically relevant sample sizes for these periods, it has been possible to assess the dietary development across the MES, not just at individual sites. Moreover, parallel examinations carried out at continuously settled individual sites have also revealed local patterns of behaviour, certain types of local structural design and specific developments in comparable conditions.

## Analysis of carbon and nitrogen isotopes for the reconstruction of human dietary habits in prehistory

In addition to collecting archaeological, archaeozoological, and archaeobotanical data, stable isotope analysis of carbon (*δ*^13^C) and nitrogen (*δ*^15^N) in collagen, the organic fraction of human and faunal bones and teeth, is a straightforward method for diet reconstruction [15,16,30,31]. Because the major nutrients carbohydrates and fats lack nitrogen, all the nitrogen found in collagen, itself a protein, is derived from dietary proteins [32]. Although carbon occurs in all major nutrients, collagen carbon is also largely derived from dietary proteins. The contribution from other nutrients is around 25% [33,34] and especially in periods of protein deficiency, carbon may also be routed from carbohydrates and fats into non-essential amino acids [35–37]. Because of their different atomic masses, light stable isotopes are fractionated during biochemical reactions. This causes enrichment of the heavier isotopes along the food chain. Between plants and the collagen of their primary consumers (herbivores) *δ*^13^C values increase by about 5 ‰ (VPDB), while the difference between collagen of species at adjacent trophic levels of a food chain is about 0.8–1.3 ‰ [38,39]. The effects of trophic level enrichments are larger in *δ*^15^N with an increase of 3–5 ‰ (AIR) for each step in the food chain [32]. Recent studies on humans estimate an even larger difference between diet and collagen of 6 ‰ [40]. From stable isotope compositions we can distinguish between consumers of marine and terrestrial ecosystems, detect positions in the food chain and changes in climate conditions, especially humidity [15,41,42]. Moreover, anthropogenic influence on soil conditions, primarily manuring, raises the *δ*^15^N values of plants [43,44]. This must be taken into consideration in diet reconstructions in order not to overestimate the contribution of meat and dairy products. In the case of *δ*^13^C, the major cause of variation is the different isotope fractionation of plants that follow the C_3_ and C_4_ photosynthetic pathways. While C_3_ plants with *δ*^13^C values of between -35 and -22 ‰ dominate in areas with temperate climates, such as our study area, C_4_ plants with *δ*^13^C values of between -12.7 and -11.4 ‰ occur in warmer environments [45]. Staple plants in the latter group include millet, maize, and sorghum, of which only millet is relevant in European prehistory, but not until post-Neolithic times [16,46,47]. Variation of carbon isotope ratios within the spectrum of C_3_ plants reflects density and extent of forest cover (“canopy effect”, [48–50]) and humidity [51,52], which is also mirrored in Neolithic collagen samples [53].

On a global scale, variation of *δ*^15^N is largely affected by temperature, precipitation, and the effects of coastal environments [54–56]. In order to account for spatial and temporal variation of the isotope baseline data, it is essential to not only analyse human bones from the contexts in question, but also comparative faunal material. The latter provides proxy data for animal-derived food sources, but also characterises typical data ranges for representatives of specific trophic levels. Because the isotopic composition of human and faunal food sources may differ remarkably, stable isotope data of botanical macro remains are equally important [57], although not always available.

Stable isotope studies which focus on the Neolithic have been conducted by several workgroups in various geographic areas of Europe; an overview and a summary of available data is given in [53]. Two studies are worth mentioning here. Based on 13 sites in southern Germany with similar cultural sequences (5,500–2,000 cal. BC), though with rather limited sample numbers, Asam and colleagues [58] observed an increase in the mean *δ*^15^N values across the period, from Early Neolithic LBK groups to the Middle Neolithic period and ending with Late Neolithic populations. An improved meat supply and a broader dietary spectrum is not postulated until the Late Neolithic, at which stage it is interpreted as evidence of a stabilisation of the existential food supply available to Neolithic settlers. Archaeological findings made at the Blätterhöhle cave site (Hagen, western Germany) point to the coexistence, locally, of different Late Neolithic population groups, which differed from a molecular genetic point of view and in terms of their subsistence strategies. Besides farmers who consumed meat and the secondary products of domestic animals, another community also lived in the area, specialising in a diet based on wild animals and freshwater fish and whose mtDNA haplogroups indicate a genetic profile typical of Mesolithic population groups [59]. Stable isotope studies often rely on selected sites and rather small datasets, or lack a long-term diachronic perspective. The study of individual sites is crucial for illustrating geographical and temporal highlights, but it does not allow the comprehensive assessment of lifestyles and subsistence and dietary habits over a specific time period, a particular region or even a (pre)historic epoch.

## Materials and methods

This study deals with archaeological skeletal material whose excavation and scientific examination was commissioned and licensed by the State Office for Heritage Management and Archaeology Saxony-Anhalt (under the directorship of Prof. Dr. H. Meller). The human remains are stored in the archive of the State Office for Heritage Management and Archaeology Saxony-Anhalt at Halle (Saale). The sample material used in the dietary reconstruction presented here, which was based on carbon and nitrogen isotope ratios in bone collagen, comprised 482 human and 109 faunal bone samples from 26 sites, spanning the central German Early Neolithic to the Early Bronze Age (S1–S4 Tables). It includes earlier published data from studies of specific time periods or sites [18,60,61]. 466 samples which complied with the quality criteria for bone collagen from archaeological contexts [62,63] were included in the data analysis. Standardised methods were applied for age and sex determination [21,64–66]; sex was only determined for juveniles and adults. The data comprise 147 males and 132 females (≥ 15 years of age at death) as well as 187 individuals of indeterminable sex, most of the latter being children (< 15 years). The age groups were defined as follows: infans I (0–6 years, n = 69), infans II (7–14 years, n = 66), juvenile (15–20 years, n = 44), adult (21–40 years, n = 183), mature (≥ 41–60 years, n = 92) and senile (≥ 61 years, n = 8). The sites extended from the northern north of the Harz uplands to the south of Saxony-Anhalt (Fig 1) and were excavated between 1991 and 2010 by the State Office for Heritage Management and Archaeology in Halle (Saxony-Anhalt). Many of the settlements and cemeteries had been settled and used several times or even continuously during the period studied. In terms of the different periods, the dataset comprises 92 individuals from the EN, 13 from the MN, 77 from the YN, 35 from the LN, 106 from the FN and 143 from the EBA (S1 Table). The high sample numbers allowed us to analyse the data by age, sex, site, cultural association and chronology whilst maintaining the statistical significance of the evidence. Besides the dataset of human individuals, there were also results from 105 samples from domestic and wild animals (S3 Table**)**. These came from burials and from settlement features (pits). The animal species examined included domestic cattle (*Bos taurus*, n = 57), aurochs (*Bos primigenius*, n = 3), sheep and goat (*Ovis aries* and *Capra hircus*, n = 19), pig (*Sus domesticus* n = 21), dog (*Canis familiaris*, n = 3), red deer (*Cervus elaphus*, n = 1) and stone marten (*Martes* spec., n = 1).

Sample preparation followed [67] with slight modifications [18,68]. Most of the human samples were taken from ribs. Where no ribs were available, we used long bones or skull fragments. For reasons of quality of preservation, we had to use different skeletal elements for the faunal samples. The surface of the bone fragments was removed with dental cutting and milling equipment and samples were demineralised in 10 ml of 0.5 N HCl (4°C, 14 days), rinsed five times with deionised water and gelatinised (48 h, 70°C, pH 3). We used Ezee-Filter separators (Elkay) to separate the insoluble portion and Amicon ultrafilters (glycerol coating removed by several washes with deionised H_2_O and 0.1 M NaOH) to concentrate long-chained collagen molecules. The collagen concentrates were afterwards frozen and lyophilised. Duplicates of 1–2 mg were weighed into tin capsules and combusted to CO_2_ and N_2_ in an elemental analyser (Vario EL III, Elementar Analytical Systems) coupled to an IsoPrime High Performance Stable Isotope Ratio Mass Spectrometer (VG Instruments). The data were normalised by means of two-point calibrations based on USGS 40 and IAEA N2 for nitrogen and CH6 and CH7 for carbon [69]. Isotope compositions are reported in *δ*-notation in per mil relative to VPDB for carbon and to AIR for nitrogen, while the measurement errors were less than ± 0.2 ‰ for nitrogen and ± 0.1 ‰ for carbon.

Concerning statistical analysis of the results, the distributions of the two stable isotope ratios were compared in the faunal samples with respect to differences between animal species and between six major time periods (EN, MN, YN, LN, FN and EBA), respectively. Distributions were visualized by stratified dot plots and statistical significance of differences was assessed using the Kruskal-Wallis-test. Group specific means values were expressed as mean +/- standard deviation (SD). In comparing animal species only groups with at least 8 animals (cattle, goat/sheep, pigs) were included in assessing statistical significance. In the human samples we assessed differences with respect to age groups, gender, time periods and archaeological cultures within time periods. Individuals of unclear cultural affiliation and the single sample of the Globular Amphora Culture (KUC) were excluded from the latter. Due to an approximately balanced distribution of age and sex in the different time periods (S1 Table) we focused on univariate analyses not adjusting for differences in other factors. Distributions were visualized by stratified boxplots or histograms, and statistical significance of differences were assessed by one-way ANOVA or t-tests. For the comparison of cultures within time periods we performed pairwise comparisons using Tukey’s method. The uniformity of the age trend across time periods was assessed by testing for an interaction between age and time period in a regression model. The joint distribution of both stable isotope ratios was visualized using scatter plots. To investigate a possible trend in the population variation with respect to the two stable isotopes, we determined the interquartile range within each culture and subjected the observed values to a meta regression with the period (numbered from 1 to 6) as covariate. The interquartile ranges were estimated as the average of the differences between the 80% and the 20%, the 75% and the 25%, and the 70% and the 30% percentile. The standard errors of the estimated interquartile ranges were determined by bootstrap.

## Results

### Sample preservation and quality criteria

The overall collagen yield of the human samples that fulfilled the quality criteria for ancient bone collagen [70,71] varied between < 0.2% and 23.7% (mean value 3.1 ± 2.3%), and of the animal bones between < 0.1% and 10.0% (mean value 2.8 ± 2.1%). The carbon content of the human collagen samples ranged from 26% to 47% (mean value 40 ± 4%) and the nitrogen content from 9% to 17% (mean value 14 ± 1%). The carbon content in the animal bone samples varied between 15% and 46% (mean value 39 ± 5%) and the nitrogen content between 5% and 17% (mean value 14 ± 2%). The atomic C/N ratio of the human samples was between 2.8 and 3.7 (mean value 3.2 ± 0.1), whilst that of the animal bones was between 3.1 and 3.6 (mean value 3.3 ± 0.1) **(**S1 and S3 Tables). A total of 466 (97%) human and 105 (96%) faunal samples fulfilled the established quality criteria [71], whilst 16 human and 4 faunal samples were excluded from further study. The latter are listed in S2 and S4 Tables.

### The faunal samples

The stable isotope ratios of the faunal samples from the central German Neolithic and Early Bronze Age varied between -23.2 ‰ (*Ovis/Capra*) and -18.8 ‰ (*Martes* spec.) with regard to *δ*^13^C and between 4.5 ‰ (*Bos taurus*) and 11.0 ‰ (*Canis familiaris*) with regard to *δ*^15^N (S3 Table). Seen across all chronological phases, the data ranges of the most common domestic animals, cattle (n = 58), sheep/goat (n = 19) and pig (n = 21), largely overlapped. The highest mean *δ*^13^C values were measured in sheep/goats (min: -23.2 ‰; max: -19.9 ‰; mean: -20.7 ± 0.7 ‰), followed by cattle (min: -23.2 ‰; max: -19.6 ‰; mean: -21.2 ± 0.6 ‰) and pigs (min: -22.5 ‰; max: -20.0 ‰; mean: -21.3 ± 0.8 ‰). Two subadult cattle (ESP_7, ESP_9) exhibited no abnormalities compared to the adult animals. The *δ*^13^C values of three probable aurochs were between -20.8 ‰ and -20.4 ‰ and therefore fell within the same range as the domestic cattle. The mean *δ*^13^C values showed significant differences between the main groups of domestic animals, i.e. cattle, sheep/goats and pigs (*H*(95) = 12.181, *p* = 0.0023) (S1 Fig).

**Fig 2:**
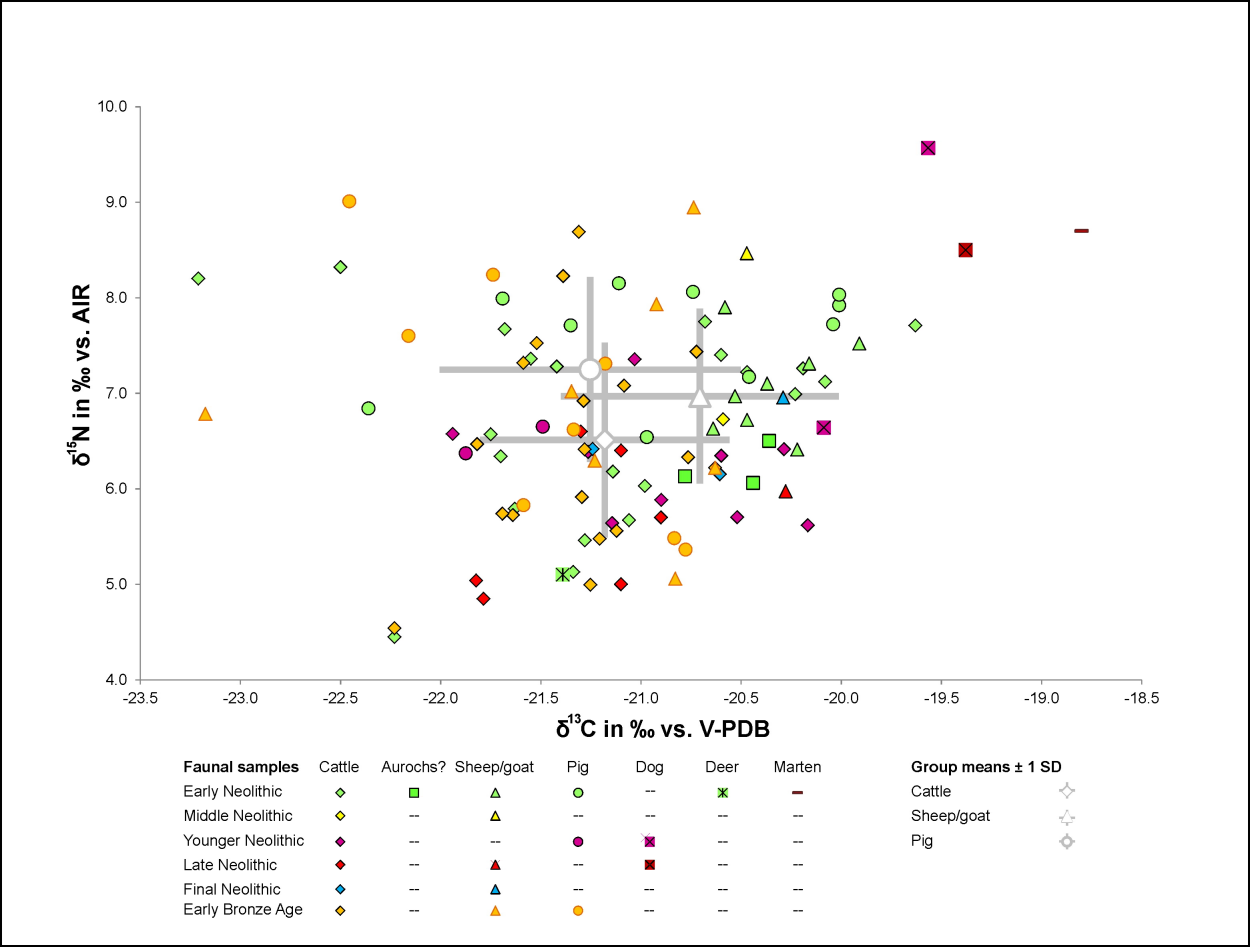
Scatter plot of *δ*^13^C and *δ*^15^N values of faunal samples. Early Neolithic (EN) = light green, Middle Neolithic (MN) = yellow, Younger Neolithic (YN) = pink, Late Neolithic (LN) = red, Final Neolithic (FN) = blue, Early Bronze Age (EBA) = orange.

Amongst the domestic animals (excluding dogs), the pigs yielded the highest *δ*^15^N values (min: 5.4 ‰; max: 9.0 ‰; mean: 7.2 ± 1.0 ‰), followed by sheep/goats (including samples that could be identified as belonging to either of those species) (min: 5.1 ‰; max: 8.9 ‰; mean: 7.0 ± 0.9 ‰) and cattle (min: 4.5 ‰; max: 8.7 ‰; mean: 6.5 ± 1.0 ‰). The ranges of *δ*^13^C values also showed significant differences between cattle, pigs and sheep/goats (*H*(95) = 9.562, *p* = 0.0084), supporting a divergence between the isotope ranges of different animal species in the food web of central Germany (Fig 2 and S1 Fig). Three samples of dogs exhibited slightly higher values with regard to both isotope ratios, thus distinguishing themselves from the herbivores (*δ*^13^C: -20.1 to -19.4 ‰; *δ*^15^N: 6.6 to 9.6 ‰). The isotope values of a single red deer sample (*δ*^13^C = -21.4 ‰, *δ*^15^N = 5.1 ‰) fell within the range of the domestic animals.

Most of the animal bones analysed could be associated with a chronological period or a particular archaeological culture. The chronological sequence of mean *δ*^13^C values of the domestic animals exhibited no discernible patterns, though differences were significant (*H*(100) = 9.242, *p* = 0.026) (Fig 2 and S1 Fig). The highest mean *δ*^13^C values dated from the Middle Neolithic (-20.5 ± 0.1 ‰), followed by the Final Neolithic (-20.7 ± 0.5 ‰) and the Early Neolithic (-20.9 ± 0.8 ‰). The lowest mean *δ*^13^C values were measured in the domestic animals from the Younger and Late Neolithic (-21.2 ± 0.5 ‰) and the Early Bronze Age (-21.4 ± 0.6 ‰). The mean nitrogen isotope values ranged between 5.7 ‰ and 7.6 ‰, and showed neither systematic changes over time nor statistical significance (*H*(100) = 5.715, *p* = 0.126) (Fig 2 and S1 Fig). Whilst the Early and Middle Neolithic domestic animals reached the highest *δ*^15^N values (7.0 ± 1.0 ‰ and 7.6 ± 1.2 ‰), the Early Bronze Age values were clearly lower (6.7 ± 1.2 ‰). The lowest mean nitrogen isotope ratios were measured in the Younger and Late Neolithic domestic animals (5.7 ± 0.7 ‰).

### The human samples

**Fig 3:**
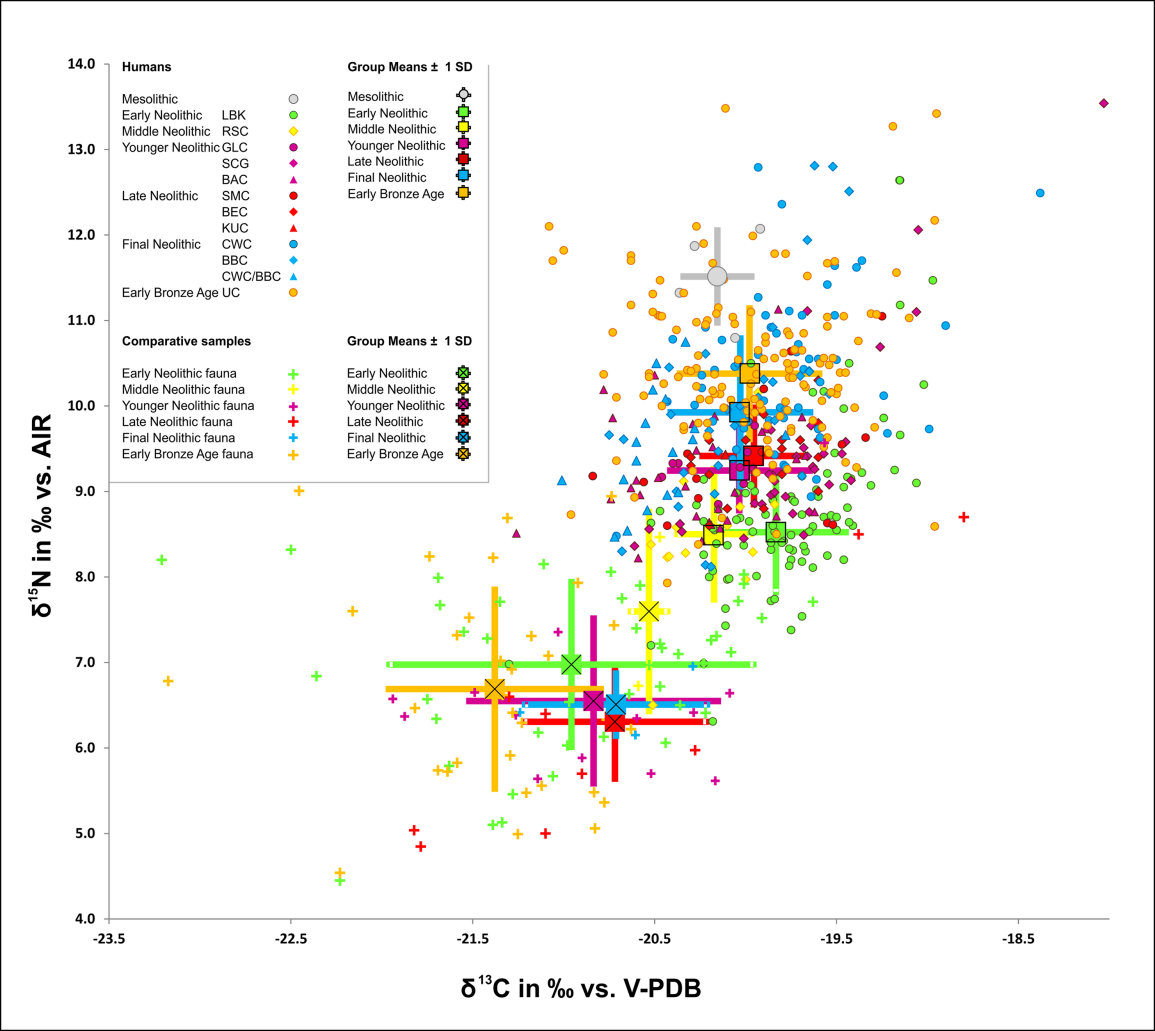
Stable isotope values of bone collagen samples from humans and animals from central Germany. Mesolithic (MESO) = grey; additional Mesolithic samples from Bottendorf, Thuringia [72]. EN = light green, MN = yellow, YN = pink, LN = red, FN = blue, EBA = orange.

**Fig 4:**
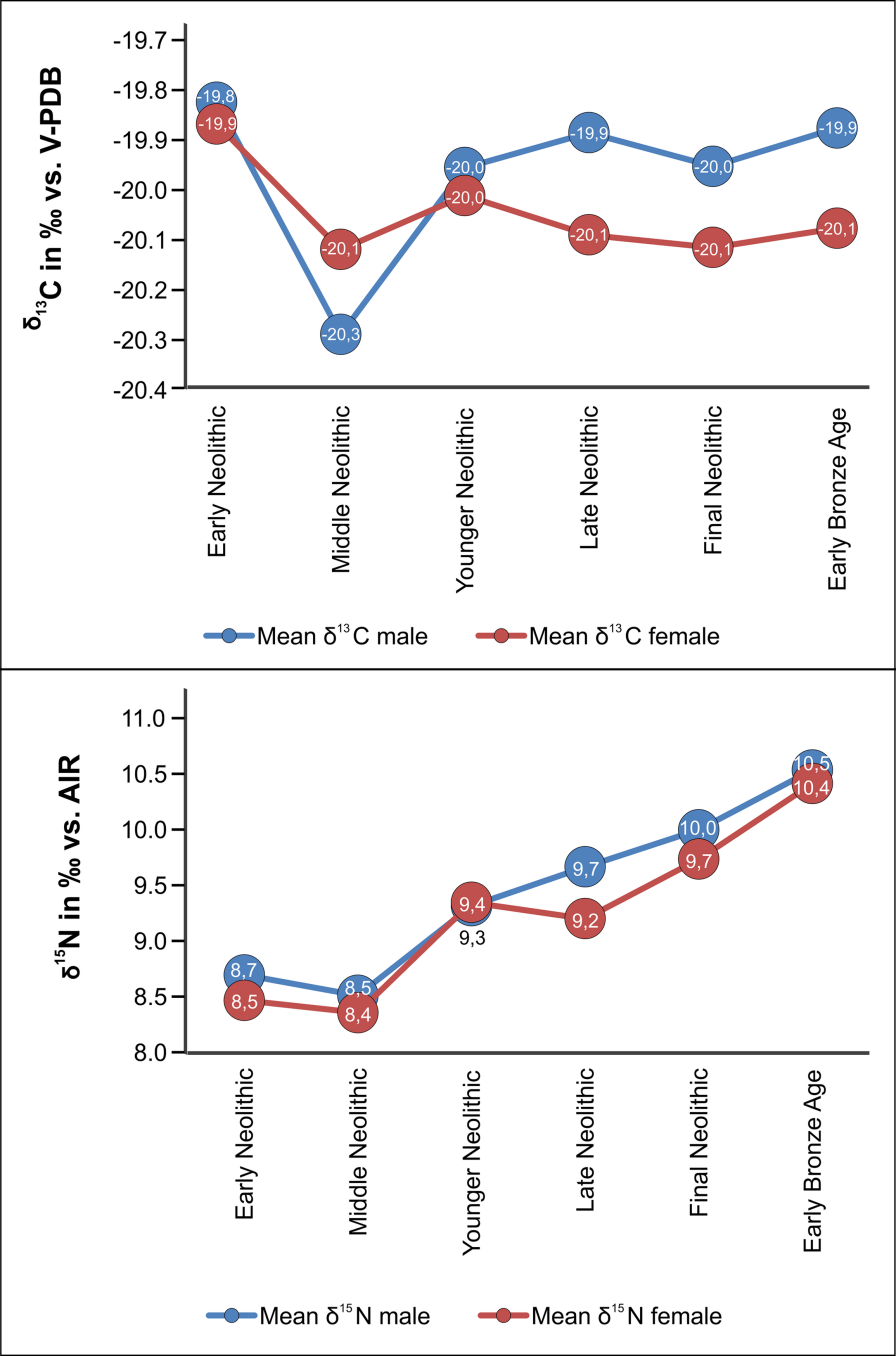
Sex-specific differences in stable carbon and nitrogen isotope values in humans.

The carbon isotope values of all human samples ranged between -21.3 ‰ and -15.4 ‰ (mean -19.9 ± 0.5 ‰), with a concentration between -21.3 and -18.9 ‰ (Fig 3). The highest value was measured in a 4–5 year-old child from Benzingerode (BENZ_46), whose Únětice Culture date is based on the position of the skeleton in the excavation area and is not secured due to a lack of diagnostic grave goods. Two infants also showed slightly raised *δ*^13^C values of -18.0 and -18.4 ‰ (SCHIEP_39 and ESP_49) (S1 Table and S2 Fig). The nitrogen isotope values overall ranged between 6.3 ‰ and 13.5 ‰ (mean 9.7 ± 1.1 ‰) (S1 Table and S2 Fig). The highest *δ*^15^N value of 13.5 ‰ was measured in a child of approximately 7–8 years (OWK_2), whilst the maximum value amongst the adults was 12.1 ‰ (ESP_15). Overall, both isotope ratios covered more than one trophic level. Both a significant sex and age difference could be demonstrated. The distribution of *δ*^15^N and *δ*^13^C for males and females shows that males had both slightly higher (*F*(1, 277) = 8.09, *p* = 0.005) average *δ*^13^C and higher (*F*(1, 277) = 6.19, *p* = 0.013) average *δ*^15^N values (*δ*^13^C = -19.9 ± 0.4 ‰, *δ*^15^N = 9.8 ± 0.9 ‰) than females (*δ*^13^C = -20.0 ± 0.4 ‰; *δ*^15^N = 9.5 ± 1.0 ‰) (Table 1 and Fig 4). The trend of marginally elevated average *δ*^13^C and *δ*^15^N values in males is almost constant throughout the Neolithic. Exceptions are found in the MN but may be attributed to low sample size. The correlations between sex and period were not significant (*F*(5, 267) = 0.49, *p* = 0.785 for *δ*^15^N and *F*(5, 267) = 0.72, *p* = 0.605 for *δ*^13^C, respectively).

**Table 1:** Chronological overview of sex-specific isotopic values. Total sample size of each chronological period, mean isotopic values of males and females (sexed juveniles included).

Period	Male					Female				
	n	Mean *δ*^13^C‰ (V-PDB)	1 SD	mean *δ*^15^N‰ (AIR)	1 SD	n	Mean *δ*^13^C‰ (V-PDB)	1 SD	Mean *δ*^15^N‰ (AIR)	1 SD
**All samples**	**147**	**-19.9**	**0.4**	**9.8**	**0.9**	**132**	**-20.0**	**0.4**	**9.5**	**1.0**
Early Neolithic	28	-19.8	0.3	8.7	0.4	29	-19.9	0.4	8.5	0.7
Middle Neolithic	4	-20.3	0.2	8.5	0.4	4	-20.1	0.3	8.4	1.5
Younger Neolithic	17	-20.0	0.3	9.3	0.4	20	-20.0	0.3	9.4	0.6
Late Neolithic	13	-19.9	0.4	9.7	0.5	12	-20.1	0.2	9.2	0.4
Final Neolithic	33	-20.0	0.4	10.0	0.8	30	-20.1	0.,4	9.7	0.9
Early Bronze Age	52	-19.9	0.4	10.5	0.6	37	-20.1	0.4	10.4	0.7

Distribution of *δ*^15^N and *δ*^13^C in the different age groups displays highly significant differences (*F*(5, 405) = 8.21, *p* < 0.0001 and *F*(5, 405) = 5.06, *p* = 0.0002, respectively). We could observe increased *δ*^15^N values in infans I and an increasing trend from infans II to senile individuals. *δ*^13^C showed a decreasing trend. We observed that the trends are present in all periods (S3 Fig). The interaction between age and period were not significant (*F*(22, 378) = 0.84, *p* = 0.673 for *δ*^15^N and *F*(22, 378) = 0.86, *p* = 0.655 *δ*^13^C, respectively).

**Fig 5:**
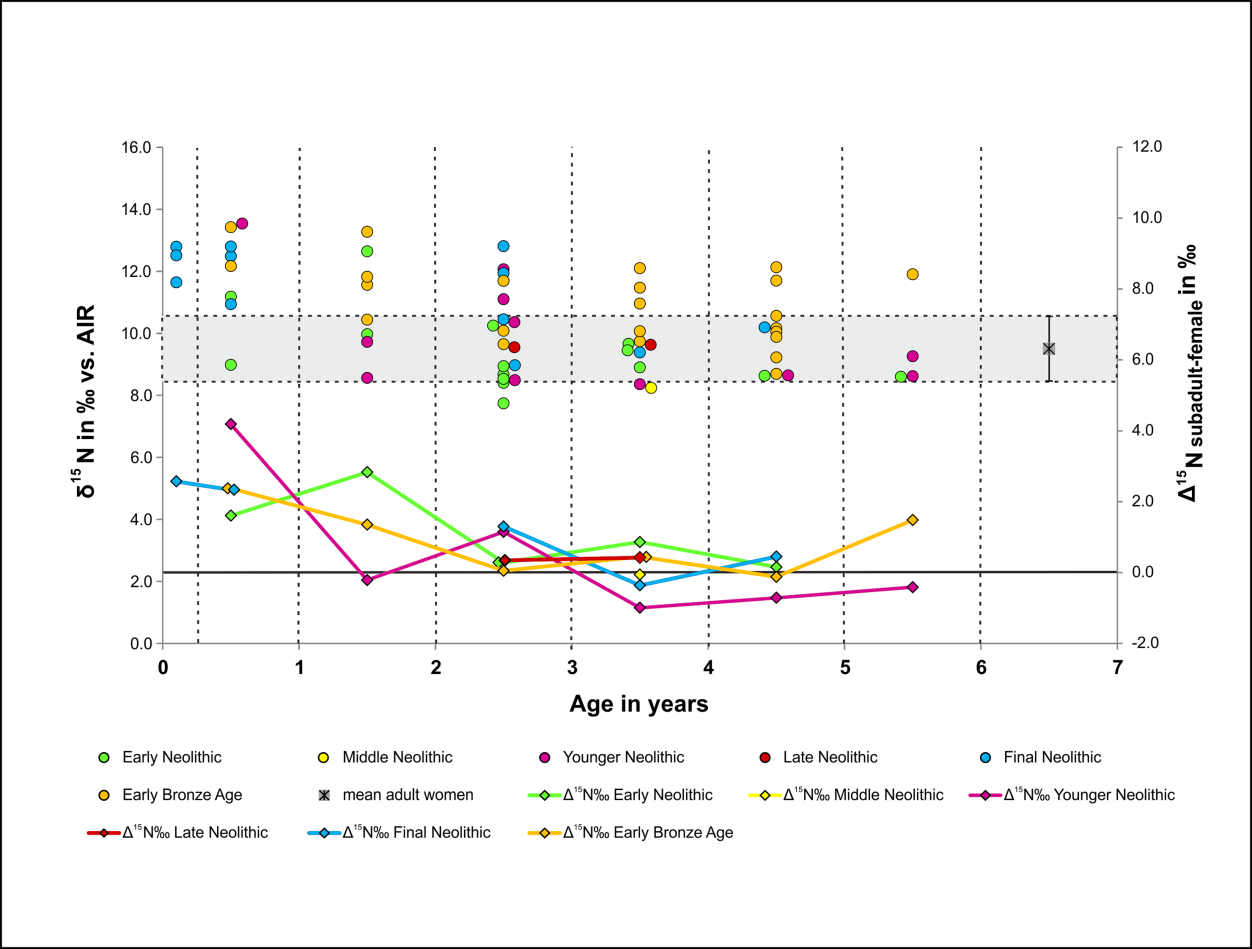
Weaning patterns. **Level of *δ***^**15**^**N in ‰ (AIR) of infans I according to age and the variable differences between the infant values and those of the females (*Δ***_**subadults-female**_**) for each time period.** EN = light green, MN = yellow, YN = pink, LN = red, FN = blue, EBA = orange.

The differences in all children of the age group infans I (mean *δ*^13^C = -19.7 ± 0.8 ‰, mean *δ*^15^N = 10.4 ± 1.6 ‰) compared to the totality of all age groups were on average +1.4 ‰ with regard to *δ*^15^N and +0.2 ‰ with regard to *δ*^13^C and occurred in all periods. A detailed analysis within the age group, comparing *δ*^15^N mean values with those of adult females, exhibited the most distinct differences amongst the newborns (+ 2.8 ‰) followed by the group of 0–1 year-olds (+ 2.4 ‰). The difference decreased to + 1.5 ‰ in the 1–2 year-old infants. From the age of 2–3 years upwards, the infants’ mean values increased only slightly, by 0.1 to 0.5 ‰. Looking at the differences between the infant values and those of the females from the same period, an increase in the *δ*^15^N values of infants below 2 years can be seen, despite the very small sample sizes for some periods, whilst the mean values of children above the age of 2–3 years ranged around those of the adult females from the same period (Fig 5). The mean *δ*^15^N values in children above the age of 3 to 4 years were sometimes slightly below those of the adult females from the same period (Fig 5). Due to the dietary differences of children of the age group infans I, the subsequent observations with regard to changes in the range of isotope values over time were based on all bone samples excluding those of this age group.

**Fig 6:**
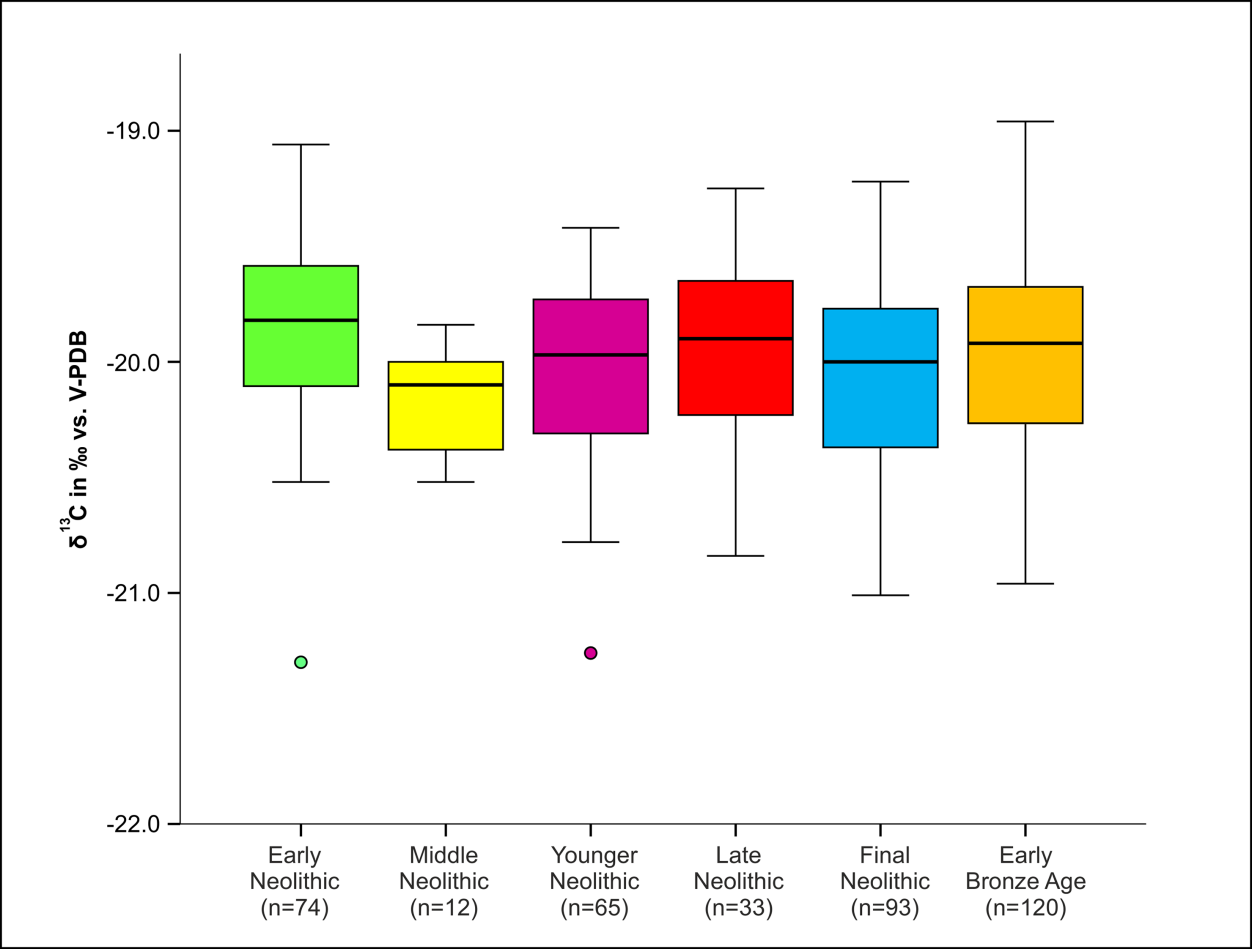
Chronological development the distribution of *δ*^13^C values according to the different archaeological periods. Numbers of individuals are displayed in parentheses.

In the case of the carbon isotope ratios, we observed no clear continuously increasing or decreasing trend in the population mean over the six time periods. Although fairly stable, however, the levels of *δ*^13^C are nevertheless statistically significantly different from each other (*F*(5, 391) = 3.60, *p* = 0.0034). The highest mean *δ*^13^C values were measured in samples from the EN (-19.8 ± 0.4 ‰), whilst the lowest occurred in the subsequent MN (**-**20.2 ± 0.2 ‰). The mean *δ*^13^C values from the other periods (YN, LN, FN, EBA) showed marginal differences only. Mean *δ*^13^C values of -20.0 ‰ were calculated for all these periods (Fig 6 and Table 2).

**Table 2:** Chronological overview of mean isotopic values. Total sample size of each chronological period, mean isotopic values of all human (infans I excluded) and animal samples. Additional information on isotopic differences of *δ*^13^C in ‰ and *δ*^15^N in ‰ between humans, domestic animals (cattle, sheep/goat, pig) and herbivores respectively (*Δ*_domestic animals-human_, *Δ*_herbivores-human_).

**Period**	**N**	**Human means**				***Δ***_**Domestic animals-human**_			***Δ***_**Herbivores-human**_	
	397	**Mean *δ***^**13**^**C‰ (V-PDB)**	**1 SD**	**Mean *δ***^**15**^**N‰ (AIR)**	**1 SD**	***Δ***^**13**^**C‰ (V-PDB)**	***Δ***^**15**^**N‰ (AIR)**		***Δ***^**13**^**C‰ (V-PDB)**	***Δ***^**15**^**N‰ (AIR)**
Early Neolithic	74	-19.8	0.4	8.5	0.7	1.1	1.5		1.1	1.7
Middle Neolithic	12	-20.2	0.2	8.5	0.8	0.4	0.9		0.4	0.9
Younger Neolithic	65	-20.0	0.4	9.2	0.5	1.0	3.0		0.8	3.0
Late Neolithic	33	-20.0	0.3	9.4	0.6	1.2	3.8		1.2	3.8
Final Neolithic	93	-20.0	0.4	9.9	0.9	0.7	3.4		0.7	3.4
Early Bronze Age	120	-20.0	0.4	10.4	0.8	1.4	3.7		1.4	3.8
**Period**	**N**	**Domestic animals**	** **	** **	** **	**N**	**Herbivore means**	** **	** **	** **
	105	**Mean *δ***^**13**^**C‰ (V-PDB)**	**1 SD**	**Mean *δ***^**15**^**N‰ (AIR)**	**1 SD**	79	**Mean *δ***^**13**^**C‰** **(V-PDB)**	**1 SD**	**Mean *δ***^**15**^**N‰ (AIR)**	**1 SD**
Early Neolithic	46	-20.9	0.8	7.0	0.9	34	-20.9	0.8	6.8	0.9
Middle Neolithic	2	-20.5	0.1	7.6	1.2	2	-20.5	0.1	7.6	1.2
Younger Neolithic	13	-21.0	0.6	6.3	0.5	9	-20.9	0.6	6.2	0.6
Late Neolithic	9	-21.2	0.5	5.7	0.7	7	-21.2	0.5	5.7	0.7
Final Neolithic	3	-20.7	0.5	6.5	0.4	3	-20.7	0.5	6.5	0.4
Early Bronze Age	32	-21.4	0.6	6.7	1.2	24	-21.3	0.5	6.6	1.2

**Fig 7:**
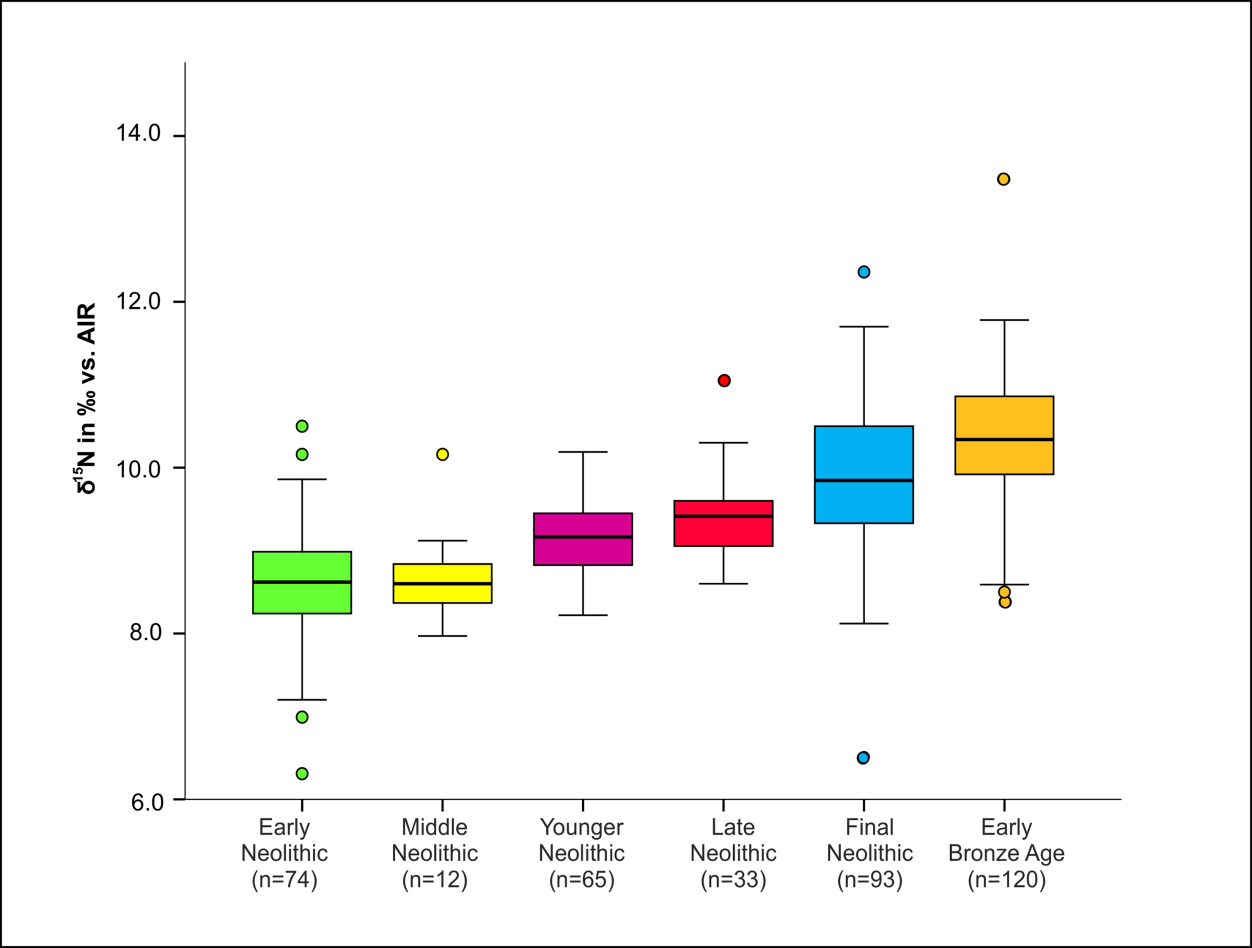
Chronological development of the distribution of *δ*^15^N-values according to the different archaeological periods. Numbers of individuals are displayed in parentheses.

Furthermore, we could observe a clear increase for human *δ*^15^N-values over time, which is highly significant (*F*(5, 391) = 67.25, *p* < 0.0001). Whilst the mean nitrogen isotope ratios of human samples from the Early and Middle Neolithic were 8.5 ± 0.7 ‰ and 8.5 ± 0.8 ‰ respectively, they rose to 9.2 ± 0.5 ‰ in the Younger Neolithic, to 9.4 ± 0.6 ‰ in the Late Neolithic, to 9.9 ± 0.9 ‰ in the Final Neolithic and to 10.4 ± 0.8 ‰, the highest level measured overall, in the Early Bronze Age (Fig 7 and Table 2).

The box plots in Figs 6 and 7 indicate a tendency to increasing population variation over time, but the trend was not uniform. A more detailed analysis based on the estimated interquartile ranges in each culture did not reach statistical significance (*F*(1,8) = 2.02, *p* = 0.193 for *δ*^15^N and F(1,8) = 0.76, *p* = 0.410 for *δ*^13^C) (S4 Fig).

**Fig 8:**
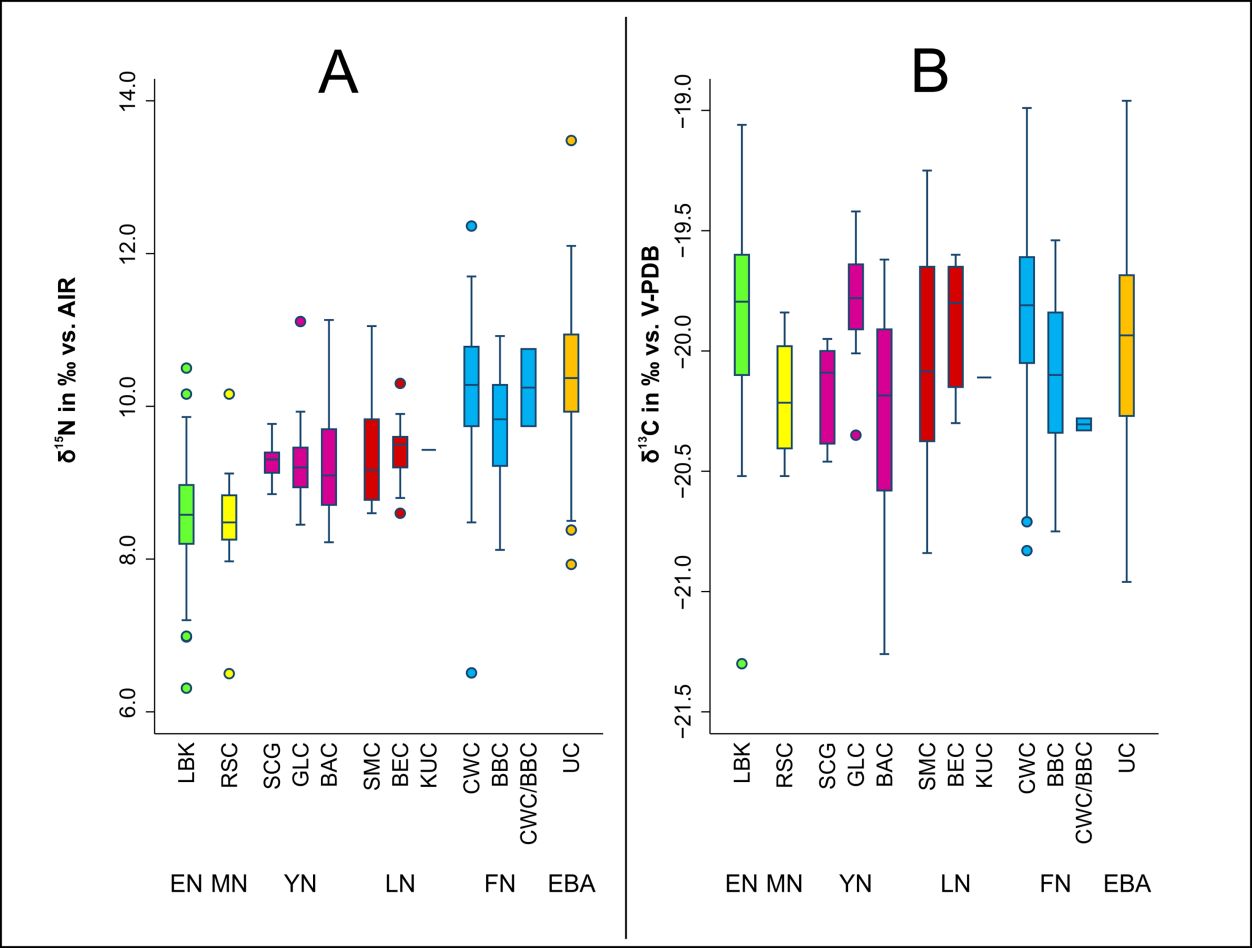
Development of archaeological cultures. Stable isotope values of bone collagen samples from humans separated according to archaeological culture (infans I exluded). EN = light green, MN = yellow, YN = pink, LN = red, FN = blue, EBA = orange.

When comparing the mean *δ*^15^N values of the individuals assigned to different archaeological cultures we observed very similar levels within each time period, except for CWC (*δ*^15^N: 10.2 ± 0.4 ‰) and BBC (*δ*^15^N: 9.7 ± 0.3 ‰) in the FN, where the only significant difference (*t* = 26.2, *p* = 0.001) was observed (Fig 8)

**Fig 9:**
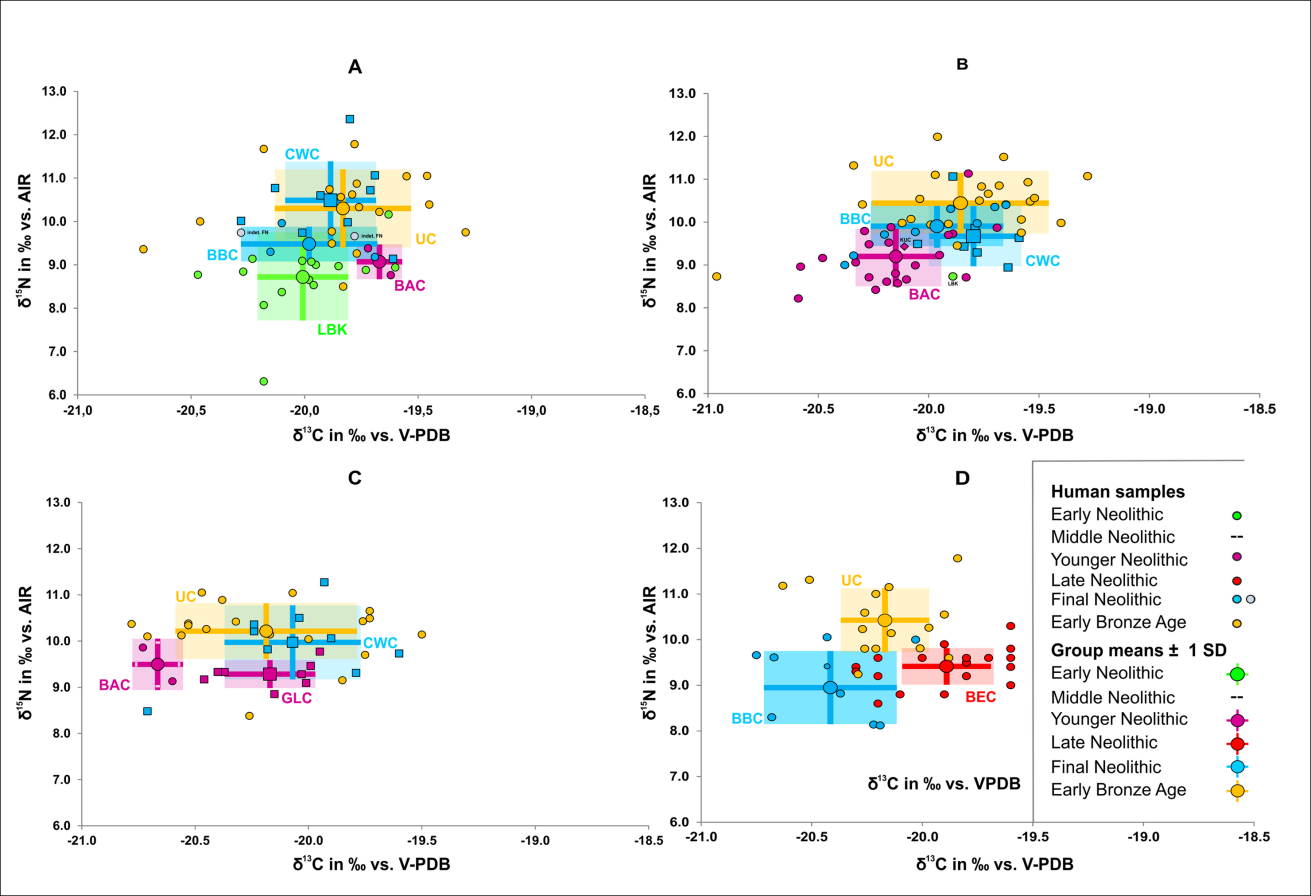
Site-specific development. Stable isotope values of bone collagen samples from humans at continually populated sites: Karsdorf with LBK, BAC, BBC, CWC, indet. LN and UC (A), Quedlinburg (B) with LBK, BAC, BBC, CWC and UC, Eulau with GLC, BAC, CWC and UC (C) and Benzingerode with BEC, BBC, UC (D) (infans I exluded). EN = green, YN = pink, LN = red, FN = blue/grey, EBA = orange.

The changes in the *δ*^15^N values observed in the overall sample throughout the course of the Neolithic were also identified at individual sites which were settled in several different periods. These sites include Karsdorf (LBK, BAC, CWC, UC), Quedlinburg (LBK, BAC, KUC, CWC, BBC, UC), Eulau (GLC, BAC, CWC, UC) and Benzingerode (BEC, CWC, BBC, UC) (Fig 9). We may therefore exclude the possibility that these changes were due to the inclusion of sites representing only one of the periods, which might have exhibited characteristics specific to the individual locality. The only two exceptions to chronologically increasing *δ*^15^N-values are UC and CWC in Karsdorf and BBC and BEC in Benzingerode. The latter is accompanied by a distinct difference in *δ*^13^C, pointing to a specific difference between these two sites. Possible reasons for these exceptions are differences in the consumption of animal protein fractions, land use strategies or the intensity of manuring, as well as such factors as local availability of resources, local animal husbandry specialisations, the bringing into use of new local areas of pasture and cultivation, and perhaps also cultural preferences.

With respect to *δ*^13^C, we detected a distinct variation within Neolithic time periods. Indeed, the following differences reached statistical significance: GLC versus SCG (*t* = 3.24, *p* = 0.005), GLC versus BAC (*t* = 8.78, p < 0.001) and BBC vs. CWC (*t* = 2.80, p = 0.007) (Fig 8). In addition to the increased mean *δ*^15^N values in the human sample over time, there was also a tendency towards increased differences between the nitrogen isotope values in the human sample and the faunal reference samples. The *δ*^15^N values of the human sample (excluding infans I) from the EN and MN were 1.5 and 0.9 ‰ respectively higher than those of the domestic animals (cattle, sheep/goats and pigs). This value rose to 3.0 ‰ in the YN and to 3.8 ‰ in the LN. A slight decrease to 3.4 ‰ was observed in the FN, however, the faunal sample was exceedingly small in this case (n = 3). The mean *δ*^15^N values of the human samples from the Early Bronze Age were 3.7 ‰ higher than those of the domestic animals (Table 2).

## Discussion

### Implications of the faunal data for landscape characterisation and animal husbandry strategies

Isotope data taken from faunal samples provide insight into specific husbandry strategies and highlight possible changes over the course of the Neolithic. They also characterise the baseline values of the potential animal-based foods for human consumption. However, it should be borne in mind that all potential forage habitats were characterised by the presence of C_3_ plants and that individual habitats and edible plants cannot always be sufficiently differentiated by means of isotope analysis. The *δ*^13^C values of all faunal samples analysed exhibited a range of values characteristic of a temperate central European climate with C_3_ plants at the bottom of the food chain, as also seen at other Neolithic sites in mainland Europe (e.g. [58,73,74]) (Fig 2). The range of variation of the data gathered from domestic fauna, indicates the use of various habitats in terms of the canopy-effect [48,50] and humidity [75,76]. Values below -22.5 ‰, considered characteristic of fodder from densely forested habitats [77], were the exception. The mean values were also higher than those measured in red deer and cattle from the Neolithic to Early Bronze Age layers at the pile-dwelling settlement Zürich-Mozartstrasse in Switzerland, which yielded evidence of a thinning out of the landscape over time [75]. Instead of showing a similar chronological trend, the data from the MES suggested that forage in non-forested habitats apparently prevailed throughout the entire Neolithic period. The carbon isotope ratios of three possible aurochs bones and of a deer as well as the results from pollen analyses [22,78] suggest that predominantly non-forested habitats were of considerable importance in this region. There is partly an overlap between the *δ*^13^C value ranges of sheep/goats and cattle which attests to the use of similar habitats for gathering fodder for ruminants. However, the higher *δ*^13^C mean value in sheep/goats and the almost complete absence of *δ*^13^C values below -21.5 ‰ which, in contrast, were quite common amongst cattle, points to an even greater importance of open tracts of land for the keeping of small ruminants as compared to cattle. The pigs also benefitted from the resources provided by various ecological niches. The relatively low *δ*^13^C values in pigs may be traced back to the consumption of mushrooms, a resource typically found in forested areas [79]. Although it is speculative, that pigs covered their daily food intake rates by fungi, we can say that low *δ*^13^C values strongly suggest low canopy food resources, likely from the forest floor.

Wide overlaps were also identified in terms of the nitrogen isotope values both in the typical herbivores, cattle and sheep/goats, and in the pigs. The mean *δ*^15^N value of the pigs was slightly higher compared to the ruminants, as one would expect to see in a species whose diet is not exclusively plant-based. The relatively modest difference implies that also pigs consumed only small amounts of animal-derived feed. The isotope ratios of the dogs attested to a more omnivorous diet. They probably benefitted from feeding on human food waste. The domestic animals in the MES exhibited much less distinctive species-specific differences overall than were found e.g. at the LBK settlement Vaihingen/Enz [57]. This can mainly be attributed to the lack of comparatively lower *δ*^13^C values due to the more open central-German landscape in the Neolithic period and Early Bronze Age. The absence of systematic differences amongst the mean isotope ratios of the different animal species over the course of the Neolithic period suggests that the environmental conditions and subsistence strategies remained fairly stable throughout the period studied here.

### Human dietary changes during the Neolithic and indications for or against a “secondary products revolution”

The analysis of the carbon and nitrogen isotope ratios of 466 burials from 26 sites in the MES dating from the Neolithic period and Early Bronze Age provided a unique insight into approximately 4,000 years of dietary history in this particular region. The most remarkable result of the diachronic study lay in the continuous increase in *δ*^15^N values in human collagen over the course of the period studied. This applied both to the mean *δ*^15^N values of the individual periods and to the differences between the *δ*^15^N values of human and faunal collagen. The changes were visible within the sites, across the sites (exceptions being CWC and UC at Karsdorf and BEC and BBC at Benzingerode) and over the entire period studied (Figs 6–9 and Table 2). The basic consistency of the C and N isotope ratios of the domestic animals over time made the trend observed in the human *δ*^15^N values appear even more significant. An overview of all isotopic values is given in Fig 3. Possible causes of the changes in the *δ*^15^N ratios in human samples include more extensive manuring, an increased consumption of meat over time and the use of milk and dairy products. In the following we discuss these different explanations.

### Changing manuring practices

In assessing the stable isotope data of human collagen, the levels of *δ*^15^N values were long seen as significant for estimating the proportions of animal protein in the prehistoric diet [32]. Animal bones, on the other hand, were used to characterise the sources of animal fodder and at the same time to study the isotope ratios of the plant-based food. However, analyses carried out on extant and archaeological cereal grains have shown that manuring fields using animal dung leads to raised *δ*^15^N values in the crops grown in those fields [44,80,81]. Whether manuring is or is not practised therefore creates a considerable difference in the isotope composition of the plants at the base of the food chain. The differences between unmanured and manured plant fodder for the animals and food plants for humans continue up the food chain and have an impact on the interpretation of the stable isotope data of bone collagen. Humans consuming cereals from manured plots exhibit higher *δ*^15^N values than humans whose food comes from unmanured fields. This means that raised *δ*^15^N values in human samples, which might appear to indicate increased consumption of animal-based food, may just as well have been caused by an increased use of animal manure. Moreover, consumption of primary or secondary products of animals pastured on fallow plots that have previously been manured could contribute to this effect. The most reliable data upon which to assess the increased importance of animal manure over time come from stable isotope analyses carried out on cereal remains themselves [44,82]. However, at the current state of research, such analyses are not, unfortunately, available for the MES. The data that are available, though, argue against an increased importance of animal manure from the Early Neolithic to the Early Bronze Age. If such an increase had taken place, one would expect it to have impacted on the *δ*^15^N values of the domestic animal bones, at least in those species assumed to partly consume agricultural products such as domestic pigs and dogs. The latter, however, did not exhibit the same diachronic increase in *δ*^15^N values that was observed for the human sample. The mean *δ*^15^N values of the Younger Neolithic to Early Bronze Age domestic animals were, in fact, lower even than those from the Early Neolithic. Even if we do not assume that animal fodder was purposefully grown on manured plots, the periodic grazing of animals on the fields after the harvest or during fallow periods may also have resulted in raised faunal *δ*^15^N values as more and more dung was added to the cultivated land over the course of the Neolithic period. Based on the chronological depth of this study and the dense Neolithic settlement of the study region one would also expect to see the former fields of abandoned settlements being used for animal grazing in later periods of the Neolithic. This way, intensive manuring practices would have had a retroactive impact on the isotope values of the faunal sample and one would assume to find them manifested as a chronological trend. Since this does not apply either, the data do not support the idea of a general man-made increase in the *δ*^15^N values in the overall food web from the early period of the food-producing economy in the MES onwards.

### Increasing consumption of animal-derived food sources: meat, milk and dairy products

Another possible explanation for the constant rise in *δ*^15^N values in the human sample is an increase in animal protein in the human diet. The growing differences between the *δ*^15^N values of domestic animals and those of humans suggest that farming communities in the LN, FN and EBA consumed significantly more animal-based food than in the previous periods of EN and MN (Table 2). A crucial question is whether these changes can be traced back solely to the increased proportion of meat in the human diet or whether they were caused by a gradual intensification in the use of secondary animal products such as milk and its dairy. If the latter was the case, the next question to be asked is at what point in time did the early farming communities begin to add their animals’ secondary products to their diet. The initial discussion on this topic dates back to Andrew Sherratt's thesis of a "secondary products revolution" [13,14,83] and continues to this day [84]. An important aspect of the discourse is the question as to whether dairy farming should be seen as a basic feature of the Neolithic lifestyle or whether this was a gradual achievement made as part of agrarian innovation. In fact, it is not possible, based on *δ*^13^C and *δ*^15^N analyses in collagen, to distinguish between protein from secondary animal products such as milk, yoghurt or cheese and protein from the direct consumption of meat [85]. Both diets are reflected in bone collagen of the consumers in a similar way. As stable isotope analyses does not allow to differentiate between the various kinds of animal protein consumption (meat versus dairy), one needs to refer to zooarchaeological, molecular genetic and lipid residue data as well as to the osteological evidence related to human health. Dental pathological studies carried out on adult skeletons from Neolithic funerary communities in the MES, which included the sites analysed isotopically for the purposes of this paper, identified almost twice the caries frequency (CF) levels for the EN and MN as for the FN and the EBA [19]. The findings were particularly clear when comparing adults of the the two largest population groups in the overall sample, i.e. the Early Neolithic LBK (CF = 66.7%; n = 62) and the Early Bronze Age UC (CF = 35.6%; n = 104), which were separated by more than 3,000 years of farming history. Differences in caries intensity (CI) between the two groups were also present, but less marked (LBK: 8.5%, teeth = 1772; UC: 5.8%; teeth = 1896). The high caries rates in the EN point to a diet rich in carbohydrates (more specifically cereals), which increases the risk of caries infection. An increase in the protein content in a diet, with a simultaneous decrease in carbohydrates, reduces the susceptibility to caries [86–88]. The protein in milk and dairy products, e.g. casein, leads to a reduction in the caries activity by reducing bacterial deposits on the dental enamel, reducing the solubility of hydroxylapatite and counteracting the demineralisation process [89–92]. Further protection against caries is provided by the abrasion of the chewing surfaces by abrasive components in the diet [93], as clearly seen in the material studied. In this case, the decrease in caries over time clearly points to a higher consumption of animal protein and simultaneous reduction in carbohydrate-rich foodstuffs. It, however, cannot necessarily be traced back solely to an increase in the consumption of dairy products. Instead, the positive impact on oral healthappears to have been based on various factors.

Other clues pointing to the consumption of animal protein are provided by the age composition and sex ratios of the animal bones, because they allow us to draw conclusions with regard to domestic animal husbandry practices. The LBK slaughter profiles argue against an extensive dairy economy, since most cattle in the MES were slaughtered before they reached 5 years of age [94,95]. Only 10% of the adult animals in the early LBK settlement Eilsleben (Börde district) were more than 5 years old [95]. The sex ratio in bovine sample, including oxen, and cows was almost equal (or “natural”) [94]. Based on the slaughter age of the animals, which matches the profile of slaughter at maturity when the greatest possible amount of meat would be available and when they could only just have started to be used to provide milk for human consumption, we may assume that the animals were kept mainly for their meat [96,97]. The same applies to the small domestic ruminants, only 20% of which were more than 4 years old when they were slaughtered [94,95]. The proportion of older animals therefore appears too low to suggest a regular production, and thus use, of milk and/or dairy products. The notion that the almost exclusive contribution of domestic animals to the human diet was meat, at least in the EN and MN, is consistent with the low human *δ*^15^N values during this periods compared to other time periods. The idea that the increase in the mean *δ*^15^N values in the YN should be interpreted as a process of economic restructuring in favour of an increase in dairy farming is questionable given the rather poor sources available. Whilst cattle were still predominant amongst the animal bones in the MES in the YN, the data on slaughter profiles are quite scarce [96]. The sources available for the use of dairy improved from the BEC in the LN onwards (Quenstedt, Mansfeld-Südharz district, and Großobringen, Weimarer Land district). Slaughter age profiles and sex ratios from these contemporary sites of that region suggest that dairy was the main use of domestic ruminants besides the exploitation for meat: traction and milk production in the case of cattle, milk and wool in the case of sheep [98].

Whether the further increase in the mean *δ*^15^N values in the FN and again in the EBA attest to the definitive establishment of dairy farming remains questionable. At any rate, the archaeozoological data available for the MES are not sufficiently conclusive.

Despite the fact that the archaeozoological data, in particular, from the MES argue against the notion that dairy farming was very important in the EN, food residue analyses carried out on pottery from other parts of Europe have yielded very early evidence in the form of milk fat residue pointing to the processing of milk and cheese-making as well as the use of dairy products [99–101]. In Germany, four sites in Saxony and Bavaria dating from the earliest to the later LBK, with a total of 87 vessels examined, yielded only a single piece of evidence of the processing of dairy products, on a strainer from the Middle LBK [99]. The evidence is surprisingly scarce, even bearing in mind that the vessels used for milk processing (such as strainers) may not have survived or may be underrepresented, and that mixing the dairy products with other foodstuffs may have masked the signals. However, particularly as a reduced-lactose dairy product at a time when lactase persistence was not yet widespread, cheese would have been a durable and easily transportable foodstuff. Analyses carried out on sieve vessels or strainers from LBK settlements on the River Vistula in Poland (5,400/5,300–4,900/4,800 cal. BC) yielded food residues from the processing of dairy products [99]. Evidence pointing to highly probable cheese-making on sieve vessels from the LBK and to the extensive use and processing of milk on beakers from the Funnel Beaker period was found at Kopydłowo (Poland) [102]. Unfortunately, researchers in this field have tended to focus on early evidence of milk processing, which has led to a lack of larger-scale studies of later Neolithic periods in Germany. Examinations of fatty acids on pottery from the settlement Arbon Bleiche 3 in Switzerland have produced evidence of milk residue as direct evidence of dairying during the LN, most likely using milk from ruminants (bovine and ovine) [103]. Based on isotope data from collagen [104], a diet with a high protein content from meat or dairy products has been postulated for CWC groups from south-western Germany, though researchers there were also unable to distinguish between the two sources of protein. The consumption of fresh milk and the consumption of dairy products such as cheese, yoghurt and kefir may also be erroneously dated to the same period and associated with lactase persistence. A newly reported genome-wide SNP dataset from 230 West Eurasians dating from between 6,500 and 300 cal. BC [9] has shown, like earlier studies [105], that no notable increase in lactase persistence in Europe appears to have occurred prior to 2,000 BC. It was and is a fact that milk is not a natural foodstuff for adult consumption, unless one is prepared to negate the numerous symptoms of lactose intolerance, including abdominal pain, bloating, flatulence, diarrhoea, asthma and others. Cultural evolution in conjunction with natural selection has made it possible for us to use milk and its secondary products as a source of protein and energy. Whilst the continuous increase in animal protein in the diet of the Neolithic populations of the MES from the LBK to the Early Bronze Age can undoubtedly partly be traced back to an intensified use of secondary animal products over the course of the Neolithic, it is difficult to estimate how great a contribution this made to the increase in *δ*^15^N values. Judging from molecular-genetic data on lactase persistence, however, the consumption of fresh milk, at least, appears to have first begun to have an impact on the protein balance of individuals around 4,000 years ago [9].

### Do possible changes correlate with the population dynamics in central Germany?

The isotope data gathered relating to the development of the dietary habits of Neolithic and Early Bronze Age populations in the MES are largely consistent with the molecular-genetically reconstructed settlement and population history of central Europe at this period, which included three events that can clearly be distinguished from the point of view of population genetics [6,7]. The first Linearbandkeramik farmers, who originally migrated from the Carpathian Basin [11] to central Europe were distinctly different from the point of view of molecular genetics from the hunter-gatherer populations of the Mesolithic (Event A). Based on the isotope data from the MES, we were able to trace clear changes in the human diet to coincide with this epochal population-genetic and economic-historical event. Recent isotope analyses carried out on Late Mesolithic individuals (5,700–5,400 cal. BC) from Bottendorf in Thuringia [72] allowed researchers to draw direct comparisons between the last hunters and gatherers and the early farmers in the MES. The study included two adults and two children of the age group infans II. The *δ*^13^C values of the human collagen ranged between -20.4 ‰ and -19.9 ‰ (mean value: -20.2 ± 0.2 ‰). The *δ*^15^N isotope values varied between 10.8 ‰ and 12.1 ‰ (mean value: 11.5 ± 0.6 ‰). Together with the available human and faunal comparative data, these values point to an opportunistic subsistence strategy, within which terrestrial food resources from fairly open and not very humid C_3_ habitats predominated. The high *δ*^15^N values, one or more trophic levels higher than the values from ungulates, show that, besides terrestrial animals (aurochs, deer, roe deer, wild horse, wild boar), fish and/or wild birds were also hunted. With the establishment of a food-producing economy by the LBK around 5,500 cal. BC, the mean *δ*^15^N value sank by c. 3‰ to the lowest mean value (8.5 ± 0.7 ‰) calculated for the entire period studied for the purposes of this paper (Fig 3 and Table 2). The big gap between the mean *δ*^15^N values in the two periods attests to the fundamental differences in the subsistence strategies. Whilst Mesolithic hunters and gatherers were more likely to use wild animals as a source of protein, the mixed diet of the early farmers in the MES was characterised by a high proportion of domestic plant-derived protein. They also consumed smaller amounts of animal primary products in the form of meat than had been the case in the Mesolithic. The constant increase in *δ*^15^N values from the LBK to the Early Bronze Age notwithstanding, the farming communities studied never yielded mean values as high as those measured in Mesolithic population groups. Even the highest *δ*^15^N mean value measured over the 4,000 year-long study period, which was reached by the UC individuals, was still c. 1.0 ‰ lower than the Mesolithic *δ*^15^N mean value (reflecting the proportion of animal protein in the diet overall).

The members of the YN and LN cultural groups (GLC, SCG, BAC, SMC, BEC, KUC) that followed the Early Neolithic LBK and the Middle Neolithic RSC were relatively homogenous genetically [6,7]. As seen from a population-genetic perspective, they experienced a reflux of hunter-gatherer lineages, brought in by the Funnel Beaker Cultures (TBC) from southern Scandinavia, which became apparent in the 4^th^ millennium BC and, in the MES, was associated mainly with the BEC (Event B). The ancestors of these groups from the north, who by this stage had also become farming communities, had at first continued the Mesolithic lifestyle in their areas of origin [6]. However, whilst the Younger and Late Neolithic increase in the mean *δ*^15^N values to 9.2 ± 0.5 ‰ and 9.4 ± 0.6 ‰ respectively marks a clear change in the dietary habits vis-à-vis the EN and MN, it cannot from a dietary point of view be traced back solely to the BEC. The massive influx of the CWC from the eastern steppes into central Europe in the FN is detectable in the MES in the first occurrences of the maternal haplogroups I, U2 and T1 [6] and also in genome-wide analyses [7]. The dietary profile once again exhibits an increase in the mean *δ*^15^N values, to 10.1 ± 1.0 ‰. The BBC, which spread somewhat later throughout north and central Europe (with the arrival of the CWC jointly making up Event C) and whose origins are presumed to have been in south-western Europe, constitutes an exception, not just from the point of view of genetics. In contrast to the general diachronic trend consisting of raised *δ*^15^N values in the cultural groups examined, the BBC exhibited a nutritional decrease in mean *δ*^15^N values to 9.7 ± 0.7 ‰. The divergence between the CWC and the BBC to be seen in their funerary rites, despite their chronological and sometimes also territorial coexistence, is thus also visible in their dietary habits. Comparative examinations of CWC sites in southern Germany have shown that their mean *δ*^15^N values were, in fact, comparable to those of the CWC in the MES (*δ*^13^C: -19.9 ± 0.6 ‰, *δ*^15^N: 10.8 ± 0.7 ‰, n = 32), despite exhibiting significant variation between and even within the sites, thus pointing to the diverging subsistence strategies of different communities [104]. The UC, which followed the CWC in the MES, bore close affinities to its forerunner in terms of its population genetics, thus supporting the hypothesis that the BBC only had a minimal genetic impact on the UC [6,7]. The close genetic links between the UC and the CWC, however, are also seen in very similar mean nitrogen values which, at 10.4 ± 0.7 ‰, were the highest in the overall sample. Moreover, a striking aspect in the evaluation of the mean *δ*^15^N values over time is a clear tendency towards rising standard deviations (S4 Fig). It is highly likely that this reflects increased social differentiation in society at the end of the Neolithic and in the Early Bronze Age. Socioeconomic advancement led to differences in status within communities and even to the formation of an elite, the differences applying to numerous facets of life, including dietary habits [60].

### Dietary differences related to age and sex

As seen in previous studies [106–108], the male samples from the MES yielded higher *δ*^13^C and *δ*^15^N values for each period and archaeological group. The maximum average differences, *Δ*_male-female_, were 0.3 ‰ for *δ*^13^C (*F*(1,277) = 8.09, *p* = 0.005) and 0.5 ‰ for *δ*^15^N in the LN (*F*(1,277) = 6.19, *p* = 0.013). In view of the varied age groups of all females studied, the lower *δ*^15^N values of the female samples are unlikely to exclusively have been due to a different nutritional physiology and nitrogen balance during pregnancy and breastfeeding [109,110] which, because of the long bone remodelling periods, may hardly had a high impact on the *δ*^15^N values of bone collagen [111]. The differences thus rather point to a higher proportion of animal protein in the diet of the males and one must question the reasons behind this. The availability of meat and dairy products may have been socially regulated, and females may have had less access to food resources with high proportions of animal protein. In the natural history of humankind, procreation and the consumption of food serve the preservation of the species. Securing the daily food intake under the prevailing environmental conditions guarantees the survival of humankind. The situation is stable if a community is able to balance production, reproduction and resources. Social and cultural differences in dietary habits are also heavily dependent on ecological and economic factors [112]. The special status of animal-based food versus plant-based foodstuffs worldwide leads to religious taboos, hierarchical distribution strategies and culture and gender-specific dietary habits. This probably reflects a distinctly gender-specific behavioural role and dietary habits handed down through the generations. It is therefore a distinct possibility that the differences in the nutritional balance between males and females are based on behaviours rooted deep in the history of humankind, which became more pronounced at the beginning of the Neolithic period as a consequence of social and economic changes [112,113]. The effects on food consumption of an unequal distribution of daily tasks and physical demands between the sexes can be seen both in traditional hunter-gatherer communities and in farming populations [114,115]. However, according to studies carried out on modern populations, physical activity does not necessarily appear to lead to lasting changes in *δ*^15^N values, provided enough time and food resources are available. Therefore, nitrogen isotope ratios are not suitable indicators for the overall physical demands made on humans or animals. If we look at the influence of sex and diet on high-performance athletes, males and females exhibit statistically significant differences in the consumption of meat (p < 0.05) but only a minimal difference in *δ*^15^N values (p = 0.075), though males generally exhibit slightly higher values. However, meat does not necessarily yield higher *δ*^15^N values nowadays than plant-derived protein [116]. Apart from clearly higher mean *δ*^15^N values in the smallest children, the comparison between the individuals from different age groups (human samples with secure age determinations) also revealed significant differences between the older age groups. From age group infans II, the *δ*^15^N values showed a slight upward trend, whilst the *δ*^13^C values remained almost constant (S3 Fig). Besides sex, the individuals’ ages apparently also played a role in their dietary habits throughout the entire Neolithic period and into the Early Bronze Age. The trends observed which, incidentally, can also be identified within the individual periods (S3 Fig), suggest that the proportions of animal protein in the human diet gradually increased in comparison with plant-derived protein.

#### Infant weaning patterns

Nitrogen isotope ratios show the shift from an input of protein by breastfeeding to an input by the intake of solid food [117–120] and are therefore useful for the study of weaning processes. As the intake of solid food, particularly of plant-based food, increases during the weaning period, the *δ*^15^N values of the overall diet gradually decrease, as seen in the children of the age group infans I in the MES. Newborns and infants under the age of twelve months exhibited distinct breastfeeding signals, but these childhood *δ*^15^N values already began to sink in the 1 to 2 year-olds and the subsequent age groups yielded similar values to those measured in the adult females of the same periods. The weaning process and the addition of solid food therefore appear to have commenced in the MES from the age of approximately 2 years (Fig 5). In the MES, unusually enriched or depleted *δ*^15^N in older children, as were observed in other studies [120–124], were noticed, too. We consider them as outliers within the normal variation. Despite the considerable length of time between the beginning of the Neolithic and the Early Bronze Age, the trends in the weaning behaviour showed close similarities throughout the entire period. All periods showed an initial decrease in the *δ*^15^N values, particularly between the first and second year, and the weaning process was usually complete between the third and fourth year. The 2 to 3 year-olds from the YN and FN, however, still appeared to be consuming a great deal of breast milk since they yielded the highest *Δ*_subadult-female_ values for *δ*^15^N (1.2 and 1.3 ‰ respectively) compared to their counterparts from the other periods. Chronologically comparable prehistoric populations throughout Europe exhibit a high variation with regard to weaning periods. The children from the LBK site Nieder-Mörlen (Hesse, Germany) were breastfed up to the age of approximately 3 years [125], whilst infants from the earlier, Mesolithic Pitted Ware Culture site Ajvide in Sweden were weaned around the age of 2 [126]. The breastfeeding periods in Neolithic and Early Bronze Age populations (KUC, CWC, UC) from south-western Poland varied within and between sites between 6 months and 3–5 years. Particularly the UC mothers in the region appear to have breastfed their children for different periods of time, which may have determined people’s individual dietary statuses from early childhood [127]. Whilst infants being weaned before the age of 18 months appears to have been a rare occurrence in prehistoric societies overall, weaning occurred slightly earlier in societies with agricultural subsistence strategies than amongst hunter gatherers. Very short breastfeeding periods of under 18 months only began to occur after the Industrial Revolution [127]. One must bear in mind, however, that factors with a physiological impact on the mother or child (e.g. malnutrition, diseases) and individual habits can prevent or interfere with breastfeeding.

## Conclusion

This study of human diet between the early stages of the farming lifestyle and the Early Bronze Age in the MES, based on carbon and nitrogen isotope analyses, is amongst the most comprehensive of its kind. Or results show that human dietary behaviour has changed significantly throughout the study period. A distinct increase in the proportion of animal protein in the human diet can be identified over time, a trend which only the people from the BBC did not follow. The results of the stable isotope analyses are consistent with epidemiological data on caries frequency, which indicate the highest proportions of carbohydrates in the human diet in the EN and the lowest in the EBA [19]. These findings may have been due to an increased consumption of either meat or dairy products. Although meat and dairy consumption cannot be distinguished by means of stable isotope data or caries frequency, molecular-genetic analyses of lactase persistence argue against an increased consumption of fresh milk [9]. However, although approximately 70% of the world population has a lactose intolerance, most of them can tolerate dairy foods or lactose-containing foods without developing symptoms [128]. It therefore comes as no surprise that the use of processed milk, i.e. dairy products, appears to have set in early on in the Neolithic period [99]. Unarguably, there was an increasing stabilisation of the supply of meat and secondary animal products throughout the Neolithic. The data dynamics overall argue against an equal availability of animal-derived protein to all sections of the various populations, which attests to early processes of specialisation, individualisation and hierarchisation. Moreover, population-genetic processes are also reflected in the development of human dietary habits. From the 4^th^ millennium BC onwards, groups moved into the MES from the north, sometimes accompanied by violence [6,29], and fundamental demographic changes took place in the FN with the arrival of CWC groups from the north-eastern steppes and the BBC from south-western Europe [6,7]. This former pastoral steppe component, in particular, may have been responsible for the fact that animal-based foodstuffs reached their highest importance in the FN and EBA. Differences in the consumption of animal-derived products between the sexes resulted in significantly lower *δ*^15^N values and less access to animal protein in females. Besides behavioural choices as to what food to consume, numerous other nutritional and gender-specific factors must certainly be taken into account when assessing the subsistence and nutritional balance of individuals. In the future, analysis of single amino acids of nitrogen and the compound-specific carbon isotope analysis of lipids and bone mineral may help providing more detailed and nuanced insight on aspects of human diet, such as protein sources in complex foodwebs, nutritional stress and disease [129–131]. They should become a standard in isotope studies and applied more often and routinely.

## Supporting information

S1 Table Summary of human samples investigated. The Mittelelbe-Saale sites are listed in chronological order, while multi-phase sites are listed numerous times. Basic information is given on geographic location, the archaeological context of the investigated individuals, individual data and isotopic data.
(XLSX)

S2 Table Summary of human samples excluded for quality reasons. (XLSX)

S3 Table Summary of faunal samples investigated. The Middle Elbe-Saale sites are listed in chronological order, while multi-phase sites are listed numerous times. Individual data of each faunal sample (ID, feature, culture, species) and isotopic data (skeletal element, collagen yield, % C, % N, C/N atomic, *δ*^13^C, *δ*^15^N) are given. For additional information on geographic location and archaeological context of the faunal remains investigated, please refer to S1 Table.
(XLSX)

S4 Table Summary of animal samples excluded for quality reasons. (XLSX)

S5 Table Summary of archaeological information. Overview of archaeological cultures of the Middle Elbe-Saale Region. Chronological period, name of culture, abbreviation, dating and distribution are listed as well as burial and settlement practices, further information and references.
(XLSX)

S1 Fig *δ*^15^N and *δ*^13^C measurements in animals stratified by species (above) and period (below). Bars display average values.
(TIF)

S2 Fig Frequency distribution of *δ*^13^C and *δ*^15^N in ‰ of human bone collagen. Curve progressions display normal distribution (n = 466).
(TIF)

S3 Fig Distribution of *δ*^13^C and *δ*^15^N in ‰ in different age groups. A = overall sample, B = archaeological periods. Small lines mark single samples, points mark outliers.
(TIF)

S4 Fig Estimated interquartile ranges of *δ*^15^N (above) and *δ*^13^C values (below) of human samples with 95% confidence intervals for each archaeological culture. The numbers are shown graphically and numerically. P-values refer to a test for trend over time.
(TIF)
