# Supplementary figures and images for: 4000 years of human dietary evolution in central Germany, from the first farmers to the first elites
Source: PLoS One. 2018 Mar 27;13(3):e0194862. doi: 10.1371/journal.pone.0194862 (PMC5870995; doi:10.1371/journal.pone.0194862)

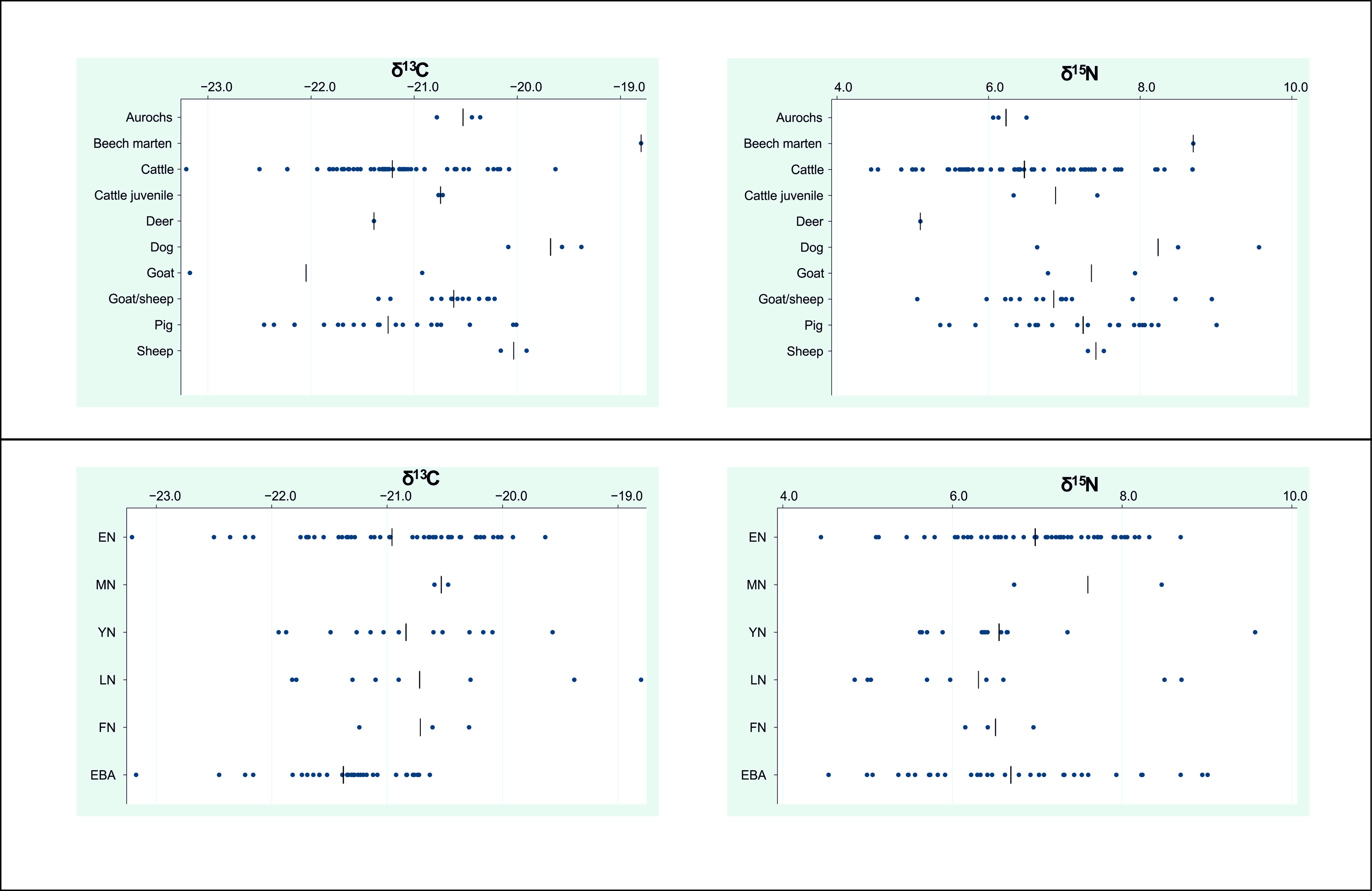

Supplement: S1 Fig — Bars display average values. (TIF) [file pone.0194862.s006.tif]

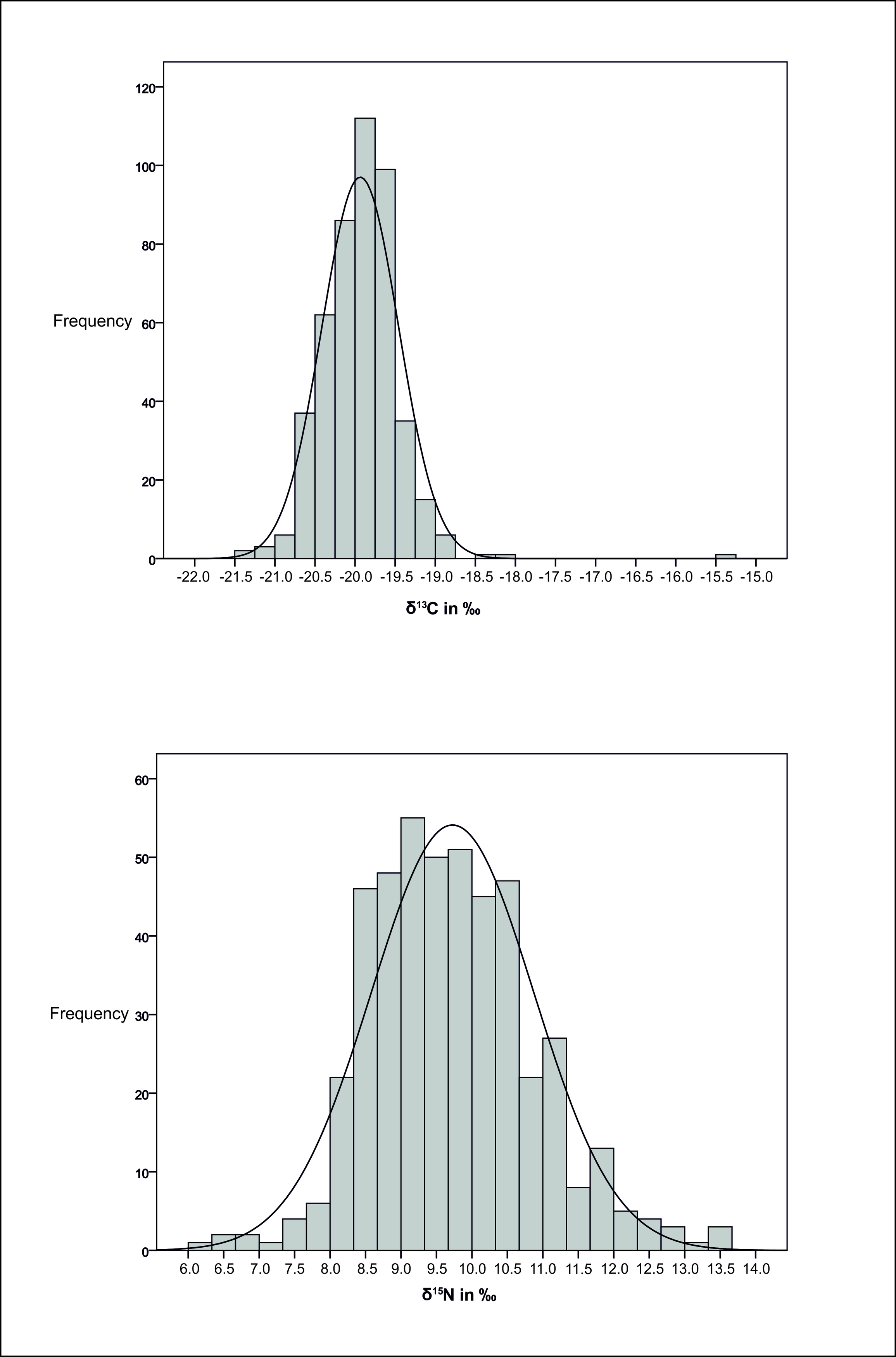

Supplement: S2 Fig — Curve progressions display normal distribution (n = 466). (TIF) [file pone.0194862.s007.tif]

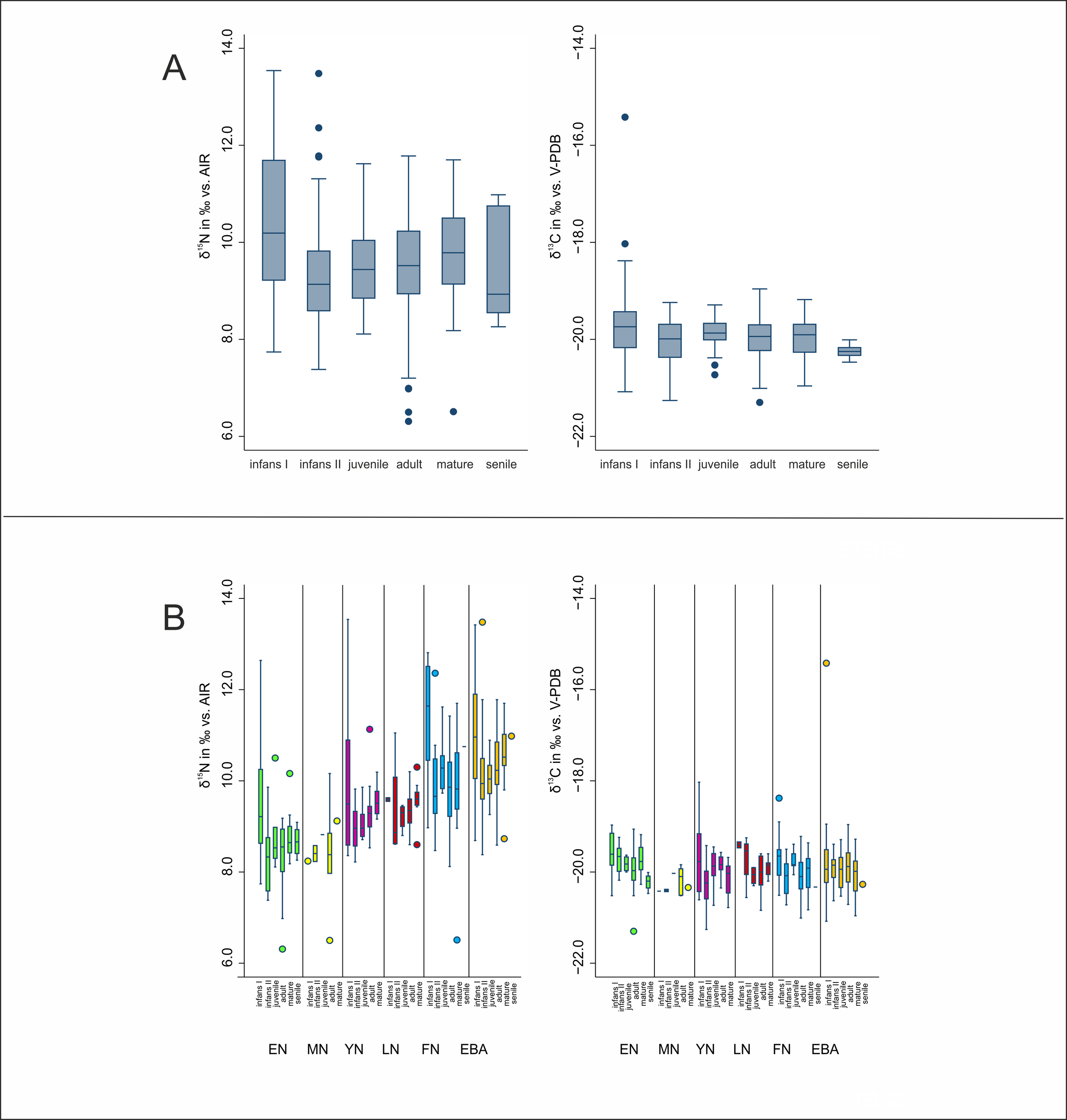

Supplement: S3 Fig — A = overall sample, B = archaeological periods. Small lines mark single samples, points mark outliers. (TIF) [file pone.0194862.s008.tif]

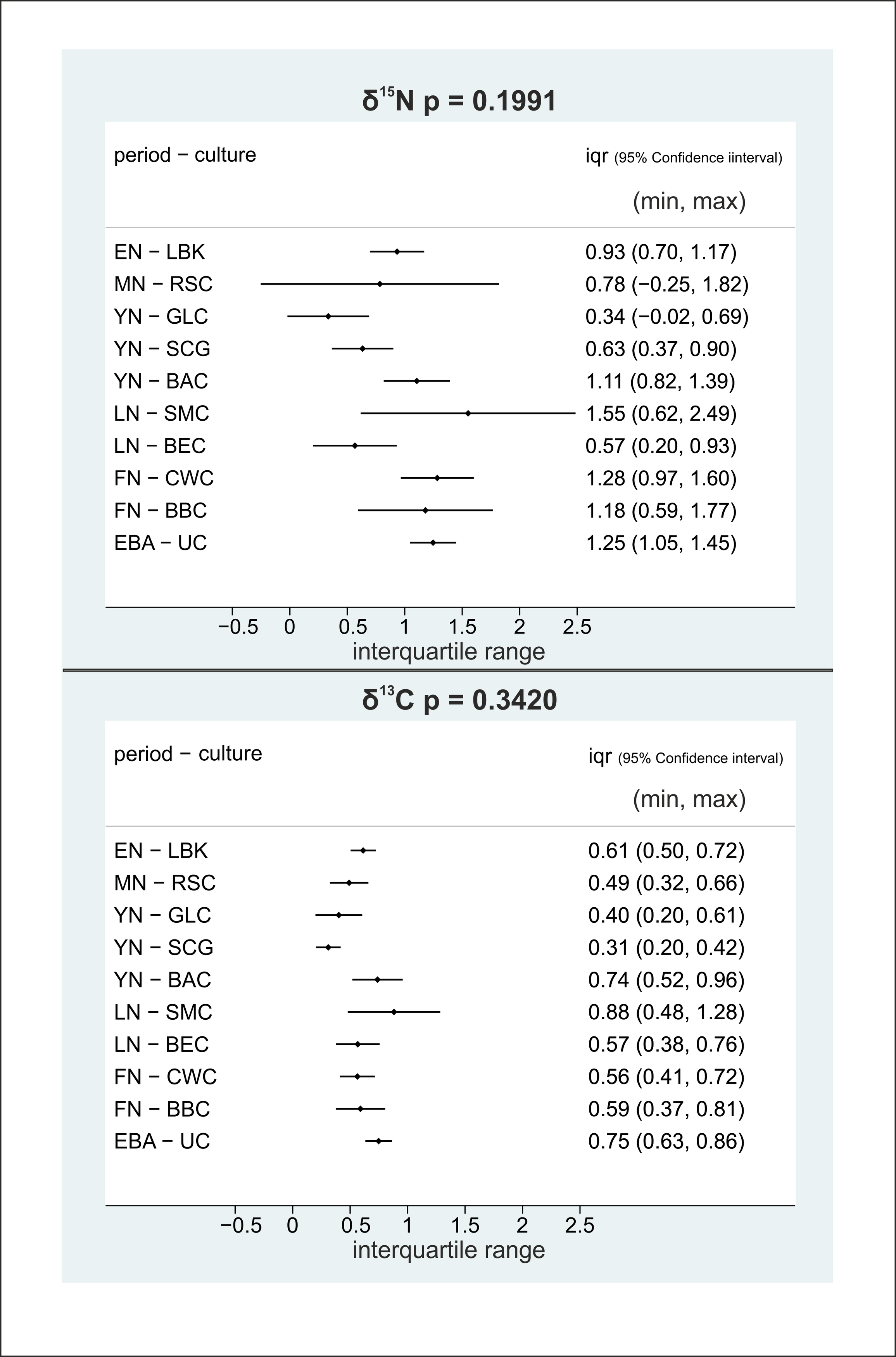

Supplement: S4 Fig — The numbers are shown graphically and numerically. P-values refer to a test for trend over time. (TIF) [file pone.0194862.s009.tif]
